# Children's Caregiving and Growth in Northwestern Tanzania: Limited Evidence That Support From Specific Caregivers Is Associated With Better Growth

**DOI:** 10.1002/ajhb.70029

**Published:** 2025-03-26

**Authors:** Anushé Hassan, David W. Lawson, Abigail E. Page, Rebecca Sear, Susan B. Schaffnit, Mark Urassa

**Affiliations:** ^1^ Department of Population Health London School of Hygiene and Tropical Medicine London UK; ^2^ Department of Anthropology University of California Santa Barbara California USA; ^3^ Centre for Culture and Evolution Brunel University London Uxbridge Middlesex UK; ^4^ Pennsylvania State University University Park Pennsylvania USA; ^5^ National Institute for Medical Research Mwanza Tanzania

**Keywords:** allomaternal care, child growth, child health, childcare, Tanzania

## Abstract

Receiving care from individuals other than one's mother (i.e., allomothering) is a universal aspect of raising children, but whether and how such care impacts children's health remains subject to debate. Existing studies in low‐income societies largely use broad proxies for caregiving behaviors rather than measuring childcare activities, which may mask variation in allomothering and, thus, its impact on children's health. Using data collected to address these limitations we measure, for 808 children under 5 years in Northwestern Tanzania: (a) Maternal residence, (b) receipt of two childcare types from seven caregivers; and (c) children's growth (height‐for‐age and weight‐for‐height). We predict that (1) allomothering will be beneficial for children's growth and (2) benefits of allomothering will be most evident within mother nonresident households. We demonstrate that children receive care from a range of allomothers, even when mothers co‐reside; and there are associations between care from different relatives. Receiving care from relatives of the same lineage tends to be positively associated, whereas care from fathers is negatively associated with care from maternal relatives. Maternal residence is not associated with child growth. We find little support for our predictions, with few and inconsistent associations between allomothering and child growth. Our findings suggest that our measures of care, while more nuanced than previous proxies, do not fully capture the complexity of caregiving. Pathways between allomothering and child growth may be further elucidated through more comprehensive care indicators, which specifically measure maternal need for help, and whether allomothering is in addition to, or substitutive of, maternal care.

## 1 Introduction

Allomothering, child care from anyone other than the mother, can consist of both provisioning of resources, material items or money as well as more intimate caregiving activities such as feeding or cleaning and help with domestic tasks (Emmott and Page 2019; Meehan 2008; Myers et al. 2021). For infants and younger children, care is most often provided by mothers. However, especially as children age, their caregivers extend far beyond mothers, encompassing fathers, grandparents, siblings, other kin and non‐kin like parents' friends and neighbors. Although studies in anthropology have often demonstrated the role of a wide range of caregivers in providing care and advice to mothers and children, global health research has traditionally had a narrower focus on the mother–child dyad or, sometimes, the nuclear family unit (Aubel 2012; Aubel et al. 2021; Sear 2021). A first step towards determining what “can best support babies and toddlers and those who care for them” (Aubel et al. 2021) is to gain a comprehensive understanding of who provides childcare, and how this relates to child wellbeing outcomes.

Who provides care to a child is dependent on context. Caregivers may adjust their care based on their presence, availability, motivation to cooperate, and the needs of the recipient mother/child (Hassan 2021; Nelson 2020; Page et al. 2019, 2022; Schacht et al. 2018; Snopkowski and Sear 2015). A key ultimate motivator for providing childcare support may be genetic relatedness (Hamilton 1964; Hrdy 2005b), and accordingly, close kin are the most common source of childcare in many populations (Crittenden and Marlowe 2008; Hrdy 1999; Ivey 2000; Kramer 2005; Meehan 2005; Sadruddin et al. 2019; Turke 1988). Nonetheless, childcare often also comes from unrelated individuals across human populations, including in small‐scale semi‐nomadic and hunter‐gatherer societies (Blurton Jones et al. 2005; Bogin et al. 2014; Crittenden and Marlowe 2008; Hrdy 2005a, 2009; Jaeggi and Gurven 2013; Meehan 2008, 2009; Meehan et al. 2013, 2016; Meehan and Hawks 2014; Starkweather et al. 2022) and high‐income, low‐fertility contexts alike (Alber 2003; Belsky 2009; Emmott and Mace 2014; Gromada and Richardson 2021; Mattison et al. 2015; Mayall 2009; Nguyen et al. 2023). These carers may be motivated by reciprocity, as has been argued to be the case among the Agta foragers in the Philippines (Page et al. 2019), alongside other direct benefits.

There is a large literature suggesting that allomothering may be associated with child health. For instance, grandmothers (particularly matrilineal) are often positively associated with their grandchildren's survival and cognitive and nutritional outcomes. Much of this research comes from studies that have used proxies of grandmaternal care, such as grandmaternal co‐residence or “presence” (Al Awad and Sonuga‐Barke 1992; Fox et al. 2010; Pope et al. 1993; Sear et al. 2000; Sear and Coall 2011; Sear and Mace 2008), though some studies have directly examined grandmaternal caregiving activities (Aubel 2010, 2012; Meehan et al. 2014; Scelza 2009; Thomese and Liefbroer 2013). However, there is variation in associations between grandmaternal care and child health, with recent research in Mexico, for example, finding no correlation between grandmaternal time spent with the mother–child dyad and child nutritional status (Vázquez‐Vázquez et al. 2021).

Patrilineal and male kin are less frequently associated with child outcomes. Again, most studies have examined the co‐residence or presence of these kin in the child's household rather than caregiving activities, and when associations are documented they tend to vary by context, associated with better child outcomes in some settings (Du et al. 2022), and with poorer outcomes in others (Beise and Voland 2002; Perry 2017; Sear and Coall 2011; Sheppard and Sear 2016). Residence with matrilineal kin is associated with better child outcomes, and patrilocal residence with poorer child outcomes, even in normatively patrilocal settings (Perry 2017). Even fathering is considered facultative (Hewlett and MacFarlan 2010; Hrdy 2006; Rosenbaum et al. 2021), and associations between fathers and child outcomes vary between settings. Father absence from households is associated with child mortality, albeit less consistently compared with associations between child mortality and maternal absence. All studies reviewed by Sear and Mace (2008) find a negative association between maternal absence and child survival, whereas a similar association for fathers is only seen in 7 out of 22 studies. Winking and Gurven (2011) also show that father desertion is associated with increased child mortality in five foraging populations, although the observed number of offspring lost to father absence is relatively small (maximum of 0.2 children). Father absence has been associated with poorer child wellbeing in the US (Sigle‐Rushton and McLanahan 2004); and with poorer child growth in Peru (Dearden et al. 2013). On the other hand, research from Mexico shows father absence is not associated with maternal ratings of child health, nor is this relationship mediated by presence of other kin (Edelblute and Altman 2018). As above, not many studies have measured fathers' caregiving activities in relation to child wellbeing. Those that have done so demonstrate mixed findings: Positive correlations between paternal caregiving and child wellbeing are documented in the Republic of the Congo and in a review of longitudinal studies in high‐income settings (Boyette et al. 2018; Sarkadi et al. 2008). In Bangladesh, Starkweather et al. (2021) show the picture is more complicated, and paternal caregiving is associated with positive child outcomes under certain circumstances. Provision of substitutive care from fathers is associated with poorer child growth; however, if this substitutive care is supplemented with care from other alloparents, children do better (Starkweather et al. 2021).

Sibling care is also subject to ecological variation, and associations between care from older siblings and younger siblings' health are mixed and generally approximated by presence (Kramer 2010). In Sear and Mace's review, the presence of siblings had a positive impact on children's survival in five out of six studies. In the Gambia, the presence of older sisters has been associated with improved survival and anthropometric status of younger children (Sear and Mace 2008; Sear et al. 2002). On the other hand, a few studies have documented negative relationships between number of older/younger siblings and children's nutritional status (Hagen and Barrett 2009; Magvanjav et al. 2013) likely due to resource competition between siblings, particularly in households with limited resources and a large number of children (Alam 1995; Lawson et al. 2012). Associations between siblings and child and maternal outcomes are also predicted to be mediated by their birth order (Kramer 2010). As above, none of these studies have specifically examined actual care provided by siblings, instead using proxy measures of care.

Much allomothering occurs while mothers are present (or perhaps only temporarily away from their children, to engage in subsistence activities or paid work, for example). But fostering—where children are separated from their parents for long periods of time—adds further evidence for the frequency and role of allomothering. A previous study in Northern Tanzania shows that father absence is associated with poorer child growth and lower household food security; however, it also finds that both mother and father absence in the context of fostering (in this case defined as residing away from both living parents) was not associated with these adverse outcomes (Lawson et al. 2017). In fact, child fostering, typically with close kin, is a common and important form of caregiving (Scelza and Silk 2014), including in the communities where this study was undertaken (Hedges et al. 2019; Urassa et al. 1997). Previous studies have demonstrated that fostered and adopted children (including orphans and those with one or both living parents) are not more disadvantaged than children living with both biological parents in terms of mortality (Mattison et al. 2015; Urassa et al. 1997) and, for those fostered by close kin, education (Hedges et al. 2019). However, fostered children have also been shown to have worse growth outcomes compared with nonfostered children, with these adverse effects lasting into adulthood (Prall and Scelza 2017; Scelza and Silk 2014).

Who provides allomaternal care is not the only factor to influence the relationship between childcare and child outcomes; the type of care, the age of the child, the reason for needing support, and household socio‐economic status and need add additional complexity. For instance, a study of Guatemalan families highlights how the intersection between source and type of support can predict different child outcomes: contact with maternal grandmothers is positively correlated with children's length/height (especially for infants), contact with grandfathers has no association, and financial help from grandfathers is associated with better nutritional outcomes for babies but poorer outcomes for older children (Sheppard and Sear 2016). In Bangladesh, Perry (2021) finds that children with deceased or divorced parents lived in lower‐income households compared to children living with both parents. Although parental death or divorce was not associated with child growth (with the exception of maternal death, which was associated with worse height‐for‐age), household income had a strong positive impact on child growth.

Despite this extensive literature, there is relatively little detailed data available on direct caregiving activities (e.g., feeding, washing, playing, supervising), particularly from non‐kin. Instead, studies often rely on proxy measures of childcare, such as the presence/absence or co‐residence of caregivers in the child's household (Nelson 2016; Sear and Coall 2011; Sear and Mace 2008) which may mask true associations between actual caregiving and children's health. Studies that do have detailed data on childcare (e.g., source, type, amount, etc.) are mostly in hunter‐gatherer societies and have small sample sizes (Crittenden 2009; Ivey 2000; Kramer and Veile 2018; Meehan 2008; Meehan et al. 2014; Page et al. 2021), or rarely examine associations between caregiving and children's outcomes (see Meehan's work and Starkweather et al. 2021 for exceptions).

To overcome these limitations, we combine parental reports of caregiving activities from a range of allomothers with a large sample of children (*n* = 808). Our childcare indicators measure whether a child received direct care from a particular caregiver at least once in the 2 weeks preceding the interview. Although children's wellbeing encompasses a wide array of factors, we focus specifically on child growth as a key measure of wellbeing. This is especially relevant as child malnutrition remains a major global health problem; in 2022, 149 million children under 5 years were estimated to be stunted and 45 million estimated as wasted (WHO 2024). In 2010, an estimated 250 million children globally were at risk of not reaching their developmental potential only because of stunting and poverty, with the largest burden falling in sub‐Saharan Africa (Lu et al. 2016). Early childhood development (including factors like children's caregiving environment, healthcare and food security) is gaining more attention from global programs and policies aiming to improve child wellbeing, for example, by the Human Capital Initiative and the Sustainable Development Goals (UNDESA 2021; World Bank 2021). In line with this, the 2018 Nurturing Care Framework provides guidance on what is considered a healthy life for a child (Britto et al. 2017; WHO et al. 2018), emphasizing “responsive caregiving” as a key component of the framework.

Our data come from two communities in Mwanza Region, Northwestern Tanzania. Our overarching hypothesis is that, all else being equal, allomaternal care will be associated with better child growth. In recognition that the source and type of allomaternal care, and the circumstances around receiving care, may result in different relationships between care and child growth, we:

1. Distinguish seven sources of allomothering: fathers, maternal grandparents, paternal grandparents, siblings, mother's siblings, father's siblings, and distant kin/non‐kin.
2. Distinguish two types of allomothering: relatively high intensity and low intensity.
3. Account for the need for allomaternal care by examining associations between caregiving and child growth for two groups of children: those who were residing away from their alive mothers and those co‐residing with their mothers.
  - We first test whether growth outcomes differ between these two groups of children.
  - We then test for associations between allomothering from each relative and child growth, determining whether these associations differ between children residing away from mothers and those co‐residing with mothers. We predict that the association will be stronger for children living away from their mothers; that is, allomaternal care will be more important for these children's outcomes.

## 2 Study Context

We collected data for this study in 2017 in Kisesa Ward in the Mwanza Region of Northwest Tanzania, within the bounds of a Health and Demographic Surveillance System (HDSS). Operated by the National Institute for Medical Research (NIMR), the HDSS has been active and collecting longitudinal demographic and health data from the local population since 1994. Our data were collected in two of seven villages in the HDSS area (rural Welamasonga and semi‐urban Kisesa), which lay on either end of a local rural–urban gradient at the time of this study. Welamasonga was primarily reliant on subsistence farming, and Kisesa was undergoing urbanization (Hedges et al. 2018). Although under‐5 mortality has declined substantially in the area over the past decade (Kishamawe et al. 2015; MoHCDGEC et al. 2016), child malnutrition continues to be a significant issue. According to the 2016 Tanzanian Demographic and Health Survey (MoHCDGEC et al. 2016) 39% of children under 5 years were stunted and 4% wasted in Mwanza Region. Food insecurity levels in the study communities were high, with half of the households sampled for this study classified as severely food insecure at the time of the survey (Hassan et al. 2019).

The majority of residents in both communities are Sukuma, an ethnic group that makes up roughly 17% of the Tanzanian population (Malipula 2014). Sukuma populations historically resided in large, scattered homes and kept sizable numbers of cattle which served as wealth; now, land holdings have reduced in size and consumer goods are more commonly used as wealth, consequently leading to a decline in herd‐keeping (Wijsen and Tanner 2002). Sukuma families also tended to have patrilineal inheritance norms and patrilocal residence after marriage, but these norms have been flexible (Wijsen and Tanner 2002). Patrilocal postmarriage residence was still visible in Welamasonga during the data collection period, with extended families at times residing in the same household and considerable distance between individual households. In contrast, in Kisesa, which was undergoing recent urbanization, households were more densely clustered, smaller household sizes were more common, and neolocal residence appeared to be on the rise. Relative to other ethnic groups in Northern Tanzania, Sukuma households are larger and tend to contain both affinal kin and fostered children (Lawson et al. 2015, 2017; Urassa et al. 1997; Varkevisser 1973). Common reasons for fostering include divorce and remarriage (i.e., fostering while one or both parents are alive) as well as the death of both parents (Kishamawe et al. 2015; Urassa et al. 1997). Previous research taking place in the same two communities found that 26% of children over 7 years were fostered (residing away from both alive parents), and another quarter resided solely with their mothers (Hedges et al. 2019). Children are most often fostered by their grandparents, particularly matrilineal kin (Urassa et al. 1997). Households can thus have diverse family structures and demographic compositions which may shape children's caregiving environments.

## 3 Materials and Methods

### 3.1 Sample and Data Collection

This study was undertaken as part of a larger project that explored marital practices and the well‐being of women and their young children (Lawson et al. 2021; Schaffnit et al. 2019). Two specific villages were sampled because of their different levels of urbanization at the time of the study (one fully rural and one semi‐urban). Within each village, households were sampled randomly and were eligible for inclusion in the study if they had a woman aged 15–35 years resident. A total of 728 households were sampled across the two villages, and household and women's surveys were carried out in all of them. Child surveys were administered in the 506 households that had a resident child under 5 years of age, leading to a total sample of 808 children (an average of 1.55 children per household; 1.78 in the village and 1.38 in the town).

The child survey was administered to either the child's biological mother, or primary guardian when the mother was not available (24% of children). The household survey recorded information on the village of residence, household composition, household size and demographic characteristics of all residents. The women's survey provided health and demographic indicators for the child's mother; and the child survey measured all child characteristics used in this paper. Children's anthropometrics (age, height, and weight) were collected as a measure of growth status for young children and as a validated indicator of malnutrition (de Onis 2006; de Onis et al. 2012; World Health Organization 2014). The child's age was provided by the survey respondent for all 808 children. Height/length and weight measurements were recorded for the majority of children (*n* = 757 for height/length; *n* = 769 for weight). All children were measured with minimal clothing and no shoes. Child height was measured to the nearest millimeter using a stadiometer for those children who were able to stand on their own; a measuring mat was used to measure the length of infants who could not stand. Henceforth, both length and height are referred to as “height.” Child weight was measured to the nearest 100 g using an electronic weighing scale on solid ground. For babies and infants who could not stand on their own we first measured the weight of their mother/guardian, and then of the mother/guardian holding the baby/infant and subtracted the former from the latter to attain the baby/infant's weight. To account for observer error, all measurements were made twice. If there was a discrepancy of 5 cm for height or 2 kg for weight, a third measurement was taken. We removed any extreme entries, and a mean height and weight were calculated for each child. Children were measured by one of five different enumerators who were rotated between both villages.

Interviews were carried out in Swahili or Sukuma by enumerators from NIMR using Open Data Kit Collect software on tablets (Hartung et al. 2010). Ethical approval was obtained from the NIMR Mwanza Lake Zone Institutional Review Board (MR/53/100/463), the Tanzanian National Ethical Review Committee (NIMR/HQ/R.8a/Vol.IX/3104), the University of California Santa Barbara Human Subjects Committee (1‐17‐0405), and the London School of Hygiene and Tropical Medicine Research Ethics Committee (13809). The focal children's biological mothers or primary guardians—who responded to the child survey—provided informed consent verbally to participate in the study and for the children's height and weight to be measured.

### 3.2 Variables

The two primary dependent variables, height‐for‐age *z*‐scores (HAZ) and weight‐for‐height *z*‐scores (WHZ), were derived using the WHO age‐ and sex‐specific growth standards (de Onis et al. 2012; WHO 1983) and calculated using macros in Stata 15 provided by the WHO (WHO and UNICEF 2019). The software automatically flags improbable *z*‐scores in the data following WHO guidelines. As such, HAZ greater than 6 or less than −6 SD and WHZ greater than 5 or less than −5 SD were considered extreme entries and removed for analysis. Following the exclusion of extreme scores, we had HAZ data for 741 children and WHZ data for 738 children. Details about these growth indicators is provided in Supporting Information: Extended materials and methods (variables).

Our key independent variables of care provision were measured from seven categories of allomothers: The child's biological father, maternal grandparents, paternal grandparents, siblings, maternal aunts/uncles, paternal aunts/uncles, and “other”. The “other” category does not include step‐parents as step‐parents were measured in a separate category which we excluded from analyses because of small sample size, but likely included distant kin not covered by the other categories (e.g., cousins) and non‐kin. Although the survey did not ask respondents to specify who “other” was, many of them referred to friends and neighbors when probed informally during data collection. As such, we have called this category “distant kin/non‐kin.” Respondents were asked if a child had received five types of caregiving, from each allomother independently, in the preceding 2 weeks: Washing, feeding, supervising (described as “watching the child passively or actively to make sure they are safe”), playing with, and providing care to the child if they had been sick (215 children [27%] had been sick in this period). For each type of care and carer the participant could respond “yes,” “no,” “don't know,” or “refuse”; this was coded into a binary variable, “yes” if the child had received that particular care from an allomother and “no” if they had not; children with a “don't know” or “refuse” response were not included in the analyses. We do not have records of the amount of allomaternal care that was provided over the previous 2 weeks (the allomother concerned might only have provided that kind of care once, or many times), or who was present or absent to provide care, therefore all allomothers are given either “yes” or “no” outcomes. The “no” category includes both living individuals who did not provide care as well as cases where that individual was dead. The five measures of care were categorized into two variables, low‐intensity care and high‐intensity care (see also Meehan 2005, 2008). Care requiring relatively low levels of energy expenditure on the part of the carer was labeled as low‐intensity care and included supervision of children (*n* = 808 for all allomothers, except for paternal aunts/uncles for whom *n* = 807 because of one “refuse” response); care requiring high levels of energy expenditure on the part of the carer was labeled as high‐intensity care and included washing, feeding/cooking for, playing with, and providing care when sick (*n* = 808 for all allomothers and care types, except for playing with the child for which *n* = 807 for maternal aunts/uncles because of one “refuse” response; and *n* = 807 for paternal aunts/uncles because of one “don't know” response). Interview questions used to measure the childcare variables are provided in Table S1.

Children's parents' vital status and residence were measured in the survey by asking if each parent was alive, and if so, did they co‐reside with the child at that time. These data were used to construct a binary variable that indicates whether the child's biological mother co‐resided with the child (*n* = 728) or was alive but did not co‐reside with the child, that is, the child was fostered (*n* = 74). Six children's mothers were not alive, and these were excluded from analysis as children with dead mothers may have had very different caregiving environments and health outcomes, and the group was too small to be analyzed on its own. Child fostering in this context may result from a number of scenarios, for example, children being born outside of marriage, being born from prior marriages and remaining with grandparents or other kin while the mother moves on with a new partner, parental divorce etc.

### 3.3 Analysis

Due to diversity in allomothering observed in other studies, we first describe variation in who provides allomaternal care to children in this population. A correlation matrix is used to test correlations between receiving care from different allomothers and identifying major categories of care arrangements. Multivariate linear regression models then test associations between maternal residence and children's HAZ and WHZ. These models compare the HAZ and WHZ of a child not co‐residing with an alive mother with the baseline of having a co‐resident mother. Finally, multivariate linear regression models examine the effect on children's HAZ and WHZ of receiving each type of care (low and high intensity) from the seven allomother categories, compared with the baseline of not receiving each type of care from that specific allomother category, with an interaction for maternal residence. We did not run multi‐level models as our sample consisted of an average of 1.55 children per household, and fixed and random effects can both be overestimated in two‐level models with unbalanced clusters and sparse observations (i.e., fewer than two) per group (Clarke 2008). We acknowledge that this may result in standard errors being biased downwards.

We ran 28 models in total as each of the seven allomother categories was modeled separately for two outcome variables (WHZ and HAZ) and two exposure variables (high‐ and low‐intensity care). As we were interested in the moderating role of maternal residence, all models included an interaction for maternal presence/absence in the household. Of the 728 children who co‐resided with their mothers, we collected HAZ data for 665 children, and WHZ data for 662 children. For the 74 children whose mothers were not resident in the household, we collected HAZ and WHZ data for 70 children.

We used directed acyclic graphs (DAGs) to illustrate the hypothesized causal relationships between variables and identify confounders to adjust for in each model using the *dagitty* package in R version 4.2.2 (Textor et al. 2016). Our conceptual framework included a number of variables to be considered as confounders in each of the models: Child's age, sex, and birth order (proxied by whether the child was their father's first child or not); number of children under 10 years residing in the same household as the child; if the child had been sick in the 2 weeks preceding the survey; household food security (proxy for household wealth); whether or not the child's household was in a rural or urban setting; if the child lived in the same household as their biological mother; and receipt of care from each of the allomothers who were not being analyzed as the exposure variable in that model (see Table S2 for list of variables). The DAG process was used to finalize which of these variables should be included in each model. Full information on the DAGs and their production can be found in Supporting Information: Extended materials and methods (analysis). This process demonstrated that in the first two models we ran, for maternal residence and each of the two child outcomes (HAZ and WHZ), we control only for child's age and whether the child was their father's first child. The DAG process next indicated that all models testing associations between allomothering and child growth (i.e., the 28 models for seven allomothers, two care‐types, and the two outcomes) control for child's age, whether the child was their father's first child, maternal presence in the household, urban/rural residence, number of under 10‐year‐olds in the household, child's recent sickness, and food insecurity. In addition to these seven control variables, the DAGs indicated that a few of the 28 models needed to control for additional variables. As such, all father care models also control for child's sex; all maternal and paternal grandparental care models also control for father care; and all maternal and paternal aunt/uncle care models also control for maternal and paternal grandparental care, respectively. An example DAG illustration is provided in Figures S1 and S2. The analysis code used to generate each of the DAGs and run all models is available at: https://osf.io/vw2cx/.

As we analyzed several models to test associations between each type of caregiving and each type of growth outcome, we ran a Holm correction for multiple testing to check for Type 1 error.

## 4 Results

### 4.1 Child, Maternal and Household Characteristics

Children had a mean HAZ of −1.6 (SD: 1.6) and a mean WHZ of 0.3 (SD: 1.4). According to the WHO categorization of “stunting” and “wasting,” 39.7% of children were stunted or chronically malnourished (low HAZ), and 4.9% were “wasted” or acutely malnourished (low WHZ). These statistics correspond to the Tanzania Demographic and Health Survey (TDHS) averages for these measurements in the Mwanza Region in 2015–2016.

On average, boys had a slightly lower HAZ and WHZ than girls (the range of HAZ and WHZ scores in our sample along with WHO cut‐offs for stunting and wasting are in Figure S3). Children aged between 1 and 3 years had the lowest HAZ compared with children in other age groups; however, children's WHZ appeared to worsen with age, indicating older children suffered more from acute malnourishment than younger ones. First‐born children had lower HAZ than later‐born children; but later‐born children had lower WHZ compared with first‐borns. Children residing away from their living mothers appeared to do worse in both outcomes compared with children residing with their mothers. Table 1 shows household, maternal and child‐level characteristics by children's HAZ and WHZ.

**TABLE 1:** Characteristics of surveyed households and children, and breakdown of children's mean HAZ and WHZ by sociodemographic indicators.

		HAZ	WHZ
		Mean (SD)	Mean (SD)
Total households surveyed—*n*	506		
Total children 0–5 years—*n*	808	−1.64 (1.58)	0.28 (1.42)
Characteristics of children			
Child's sex—*n* (%)			
Girl	397 (49.13)	−1.56 (1.43)	0.30 (1.35)
Boy	411 (50.87)	−1.73 (1.71)	0.26 (1.49)
Child's age—*n* (%)			
0–1 years	165 (20.42)	−1.04 (2.12)	0.49 (2.08)
1–2 years	156 (19.31)	−2.07 (1.35)	0.42 (1.42)
2–3 years	165 (20.42)	−1.82 (1.65)	0.32 (1.13)
3–4 years	177 (21.91)	−1.57 (1.28)	0.18 (1.29)
4–5 years	145 (17.95)	−1.70 (1.22)	−0.01 (0.98)
Father's first child—*n* (%)			
Yes	167 (21.63)	−1.89 (1.66)	0.50 (1.31)
No	605 (78.37)	−1.58 (1.56)	0.23 (1.44)
Characteristics of living mothers			
Residence—*n* (%)			
Mother co‐resident	728 (90.1)	−1.62 (1.58)	0.31 (1.46)
Mother not co‐resident	80 (9.90)	−1.85 (1.60)	0.01 (0.98)
Age—*n* (%)			
15–19 years	41 (6.69)	−1.56 (1.71)	0.94 (1.37)
20–24 years	170 (27.73)	−1.91 (1.65)	0.39 (1.58)
25–29 years	186 (30.34)	−1.59 (1.60)	0.38 (1.33)
30–35 years	216 (35.24)	−1.46 (1.51)	0.06 (1.40)
BMI—*n* (%)			
Underweight (BMI < 18.5)	50 (9.43)	−2.00 (1.13)	−0.25 (1.55)
Normal weight (BMI 18.5–24.9)	383 (72.26)	−1.59 (1.67)	0.30 (1.47)
Overweight (BMI 25–30)	72 (13.58)	−1.27 (1.68)	0.57 (1.38)
Obese (BMI > 30)	25 (4.72)	−1.62 (1.40)	0.15 (1.08)
Height—mean (SD; min, max)	1.59 (0.06; 1.44, 2.07)		
Characteristics of households			
Residence—*n* (%)			
Town	294 (58.10)	−1.59 (1.58)	0.27 (1.50)
Village	212 (41.90)	−1.70 (1.58)	0.28 (1.35)
Food insecurity—mean (SD; min, max)	10.26 (7.6, 0, 27)		
Household size—mean (SD; min, max)	7.67 (3.10; 3, 25)		
Under‐5s in HH—mean (SD; min, max)	1.75 (0.98; 1, 7)		

### 4.2 Receipt of Childcare

**FIGURE 1:**
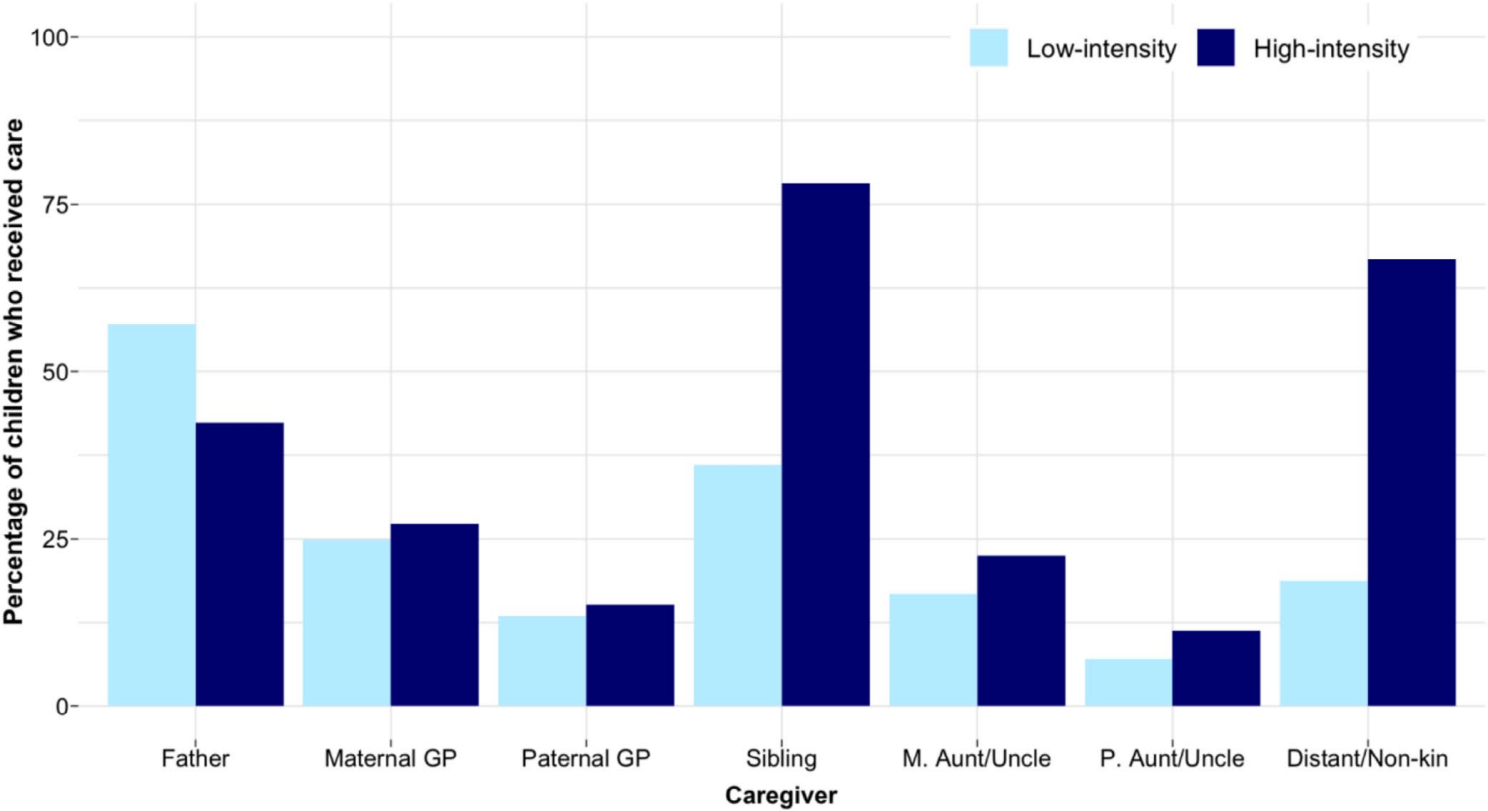
Percentage of children (*n* = 808) receiving low‐ and high‐intensity care from each caregiver.

The mean number of allomothers per child was 2.9 (SD = 1.2, min = 0, max = 7). Of the total 808 children, the majority had received care from at least one allomother (98.5%), with only 12 children (1.5%) receiving care from only their mothers (and no other caregiver). Children received care most commonly from fathers, siblings, and distant kin/non‐kin, followed by maternal grandparents (Figure 1). All allomothers, except fathers, provided more high‐intensity care (washing, feeding, playing, and caring when sick) than low‐intensity care (supervision), and fathers provided more low‐intensity care than any other allomother. More than half of the children received high‐intensity care from siblings and distant kin/non‐kin, but fewer received low‐intensity care from either of these allomothers.

**FIGURE 2:**
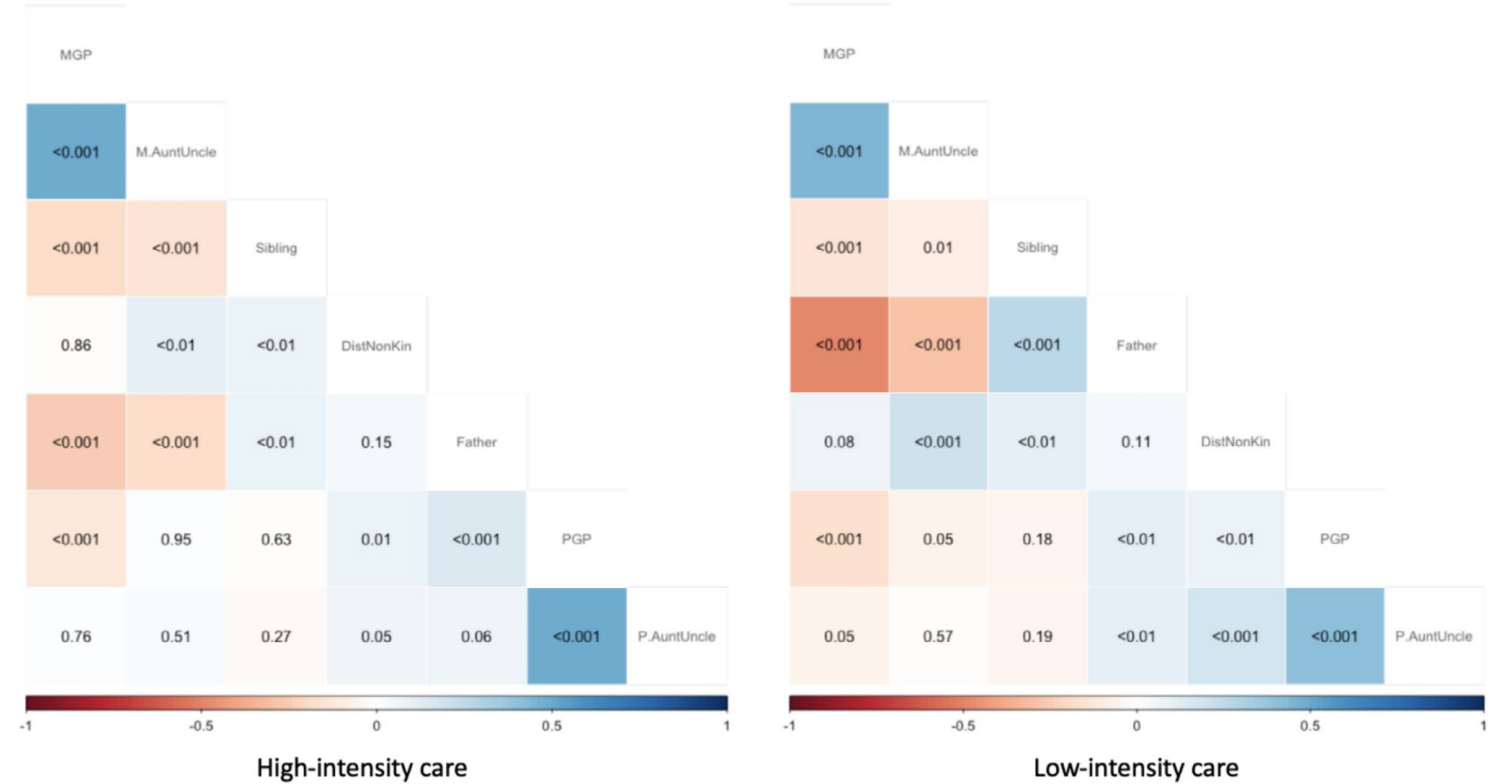
Correlation matrices showing associations between care provided to children from the seven caregiver categories for high‐ and low‐intensity care separately. A blue‐shaded cell indicates positive association between care provided by the two categories of caregivers, a cell shaded red indicates a negative association, and white cells pertain to neutral/no associations. *p* values are shown in the cells. DistNonKin, distant kin or non‐kin; M.AuntUncle, maternal aunt/uncle; MGP, maternal grandparents; P.AuntUncle, paternal aunt/uncle; PGP, paternal grandparents.

Correlation matrices (Figure 2) broadly show that care from children's mother's relatives is positively correlated (children who receive care from maternal grandparents are also likely to receive care from maternal aunts/uncles). Similarly, care from children's father's relatives is positively correlated. Children who receive care from fathers are also likely to receive care from father's relatives but are less likely to receive care from mother's relatives. Sibling care tends to be positively correlated with paternal care but negatively correlated with care from both maternal and paternal grandparents and aunts/uncles. Finally, care from distant kin/non‐kin tends to be positively correlated with care from all other individuals.

**FIGURE 3:**
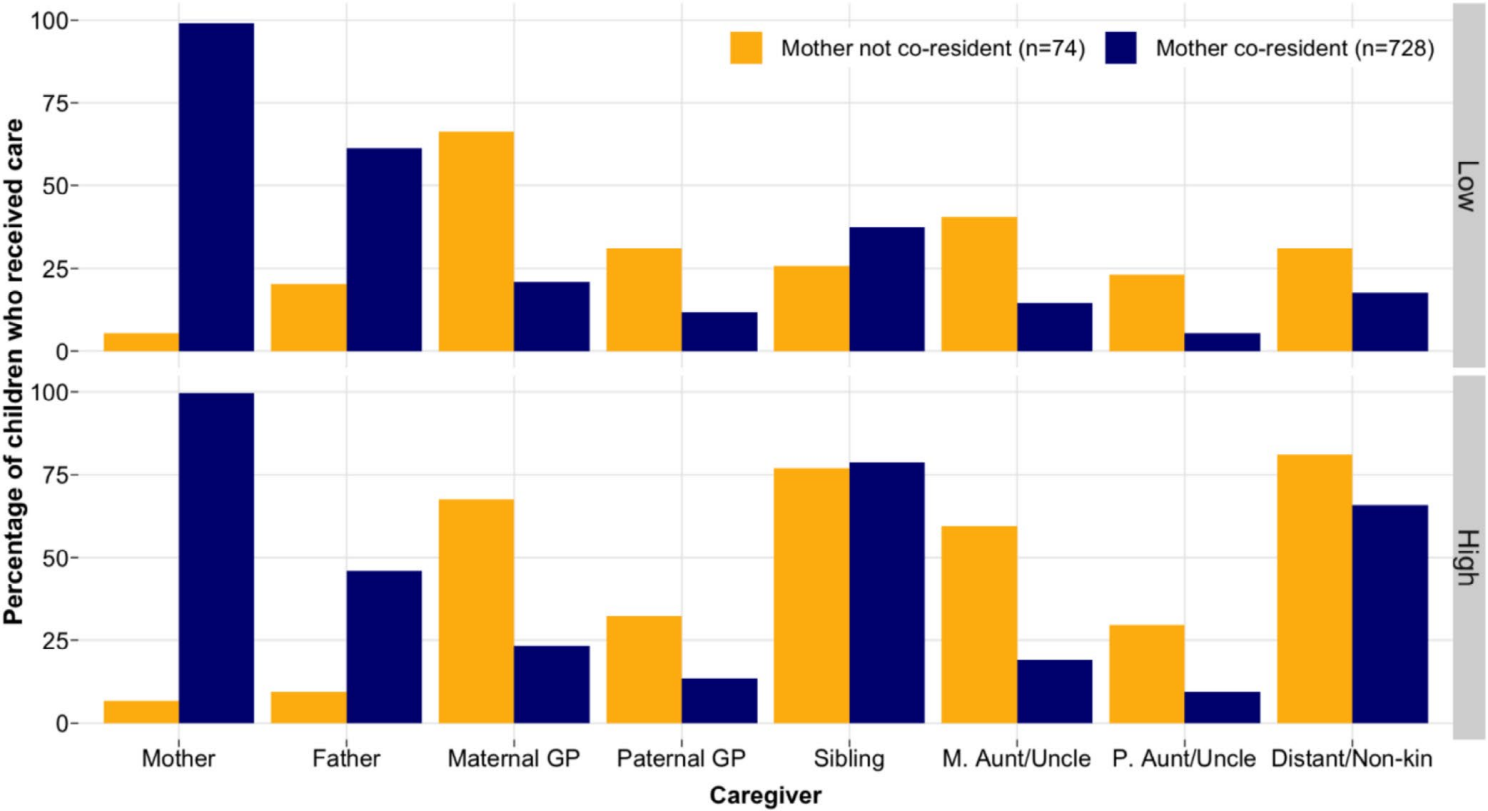
Care provided from alive mothers (*n* = 802) and seven caregiver categories to children who co‐resided with their biological mothers (*n* = 728) compared with children residing without their living biological mothers (*n* = 74).

The majority of children residing with their mothers had received care from them in the 2 weeks preceding the survey; but very few mothers who were alive but nonresident provided care (Figure 3). More fathers and siblings provided care in households where the mother was co‐resident compared with mother nonresident households. This was likely at least partly driven by the fact that of the 74 children who did not reside with their mothers, 51 (65%) also did not have co‐resident fathers (*n* = 48 for nonresident fathers and *n* = 3 for dead fathers). Conversely, all other allomothers provided more care to children who lived without their mothers compared with children with co‐resident mothers. Patterns were similar for both low‐ and high‐intensity care.

### 4.3 Maternal Presence and Child Outcomes

Child growth was not related to maternal co‐residence, contrary to predictions. Although the point estimates in models predicting HAZ and WHZ were both negative, in line with predictions, when mothers were absent from a household, the 95% confidence intervals were wide and associations were relatively small for both HAZ (*b* = −0.110, 95% CI [−0.550, 0.330], *p* = 0.624) and WHZ (*b* = −0.199, 95% CI [−0.590, 0.191], *p* = 0.317). Full models are available in Table S3: Extended results.

### 4.4 Allomaternal Care and Child Outcomes

**FIGURE 4:**
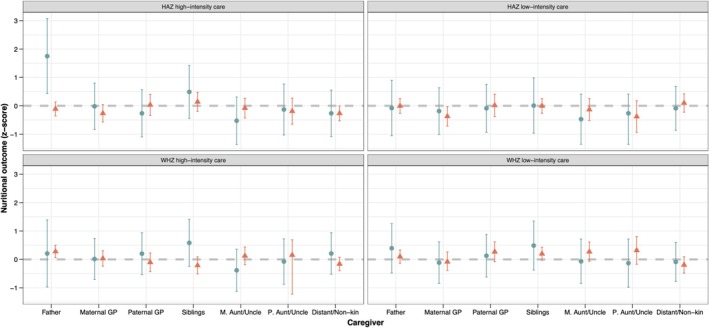
Predicted HAZ and WHZ scores with 95% confidence intervals from regression analyses of allomaternal care and child outcomes (HAZ—top row, WHZ—bottom row, high intensity care—left column, low intensity care—right column). Circle (blue) point estimates are for nonresident mothers, triangles (red) for co‐resident mothers. GP, grandparents, M.Aunt/Uncle, maternal aunts/uncles, P.Aunt/Uncle, paternal aunts/uncles.

We find very little support for our prediction that children who received allomaternal care, from a range of different carers, have improved HAZ and WHZ compared with those who did not receive care (Figure 4, full models in Tables S4–S10). The strongest association seen is between high‐intensity care from fathers and children's height‐for‐age, with care from fathers associated with a 1.75 point increase in children's HAZ (95% CI [0.428, 3.076], *p* = 0.01); this relationship is driven by paternal care in the absence of mothers (interaction term *b* = 1.863, 95% CI [0.518, 3.209], *p* = 0.007; see Figure 4 for predicted values). However, it is worth noting that this result pertains to a very small subsample of children: Of the 74 children with nonresident mothers, only 20% (*n* = 15) had received care from their fathers. No other interaction was significant in the analysis, indicating that maternal presence is only an important moderating factor for paternal care. Paternal high‐intensity care was also positively correlated with children's WHZ when mothers were present in the household, but the association was smaller (*b* = 0.280, 95% CI [0.061, 0.500], *p* = 0.012).

The remaining models reveal a mixed picture with primarily null results. We find no associations for siblings, paternal grandparents, maternal aunts/uncles, and paternal aunts/uncles for both outcomes, both types of care, and regardless of maternal residence. We do find that, contra predictions, low‐intensity allomothering from maternal grandparents was negatively associated with HAZ (*b* = −0.371, 95% CI [−0.714, −0.028], *p* = 0.034) when mothers were present (but the interaction was nonsignificant, indicating maternal presence was not important). As seen in Figure 4, the point estimates for care from maternal grandparents hovered on the line or below it, indicating a negative trend. Finally, high‐intensity care from distant kin/non‐kin was also negatively associated with children's HAZ, but the association was very small (*b* = −0.265, 95% CI [−0.528, −0.003], *p* = 0.048). As with maternal grandparents, the point estimates for distant kin/non‐kin tended to be negative. Receiving low‐intensity care was not associated with better weight‐for‐age *z*‐scores for any allomother (Figure 4). To account for associations between a child's being sick and their receipt of care, we additionally examined differences in allomaternal care provision between sick and healthy children. Results suggest no difference in the receipt of care provision between sick and healthy children (see Figure S4: Extended results).

### 4.5 Holm Correction

The Holm correction for multiple testing pushed the *p* values for all models above 0.05. The corrected *p* value (*p*Holm) for each model is provided in Tables S4–S10.

### 4.6 Discussion

Across allomother categories, types of allomaternal care, and two measures of child growth, we find scant evidence in support of our hypothesis that allomothering is associated with improved child outcomes. Nor do we find evidence that residing in mother‐absent households is associated with poorer growth outcomes. When examined as a whole, our results suggest a null relationship between allomaternal care from specific allomothers and child growth. This suggests that, despite the diversity of childcare arrangements present, children do not appear to suffer or benefit from any particular arrangements. Possibly, families may be ensuring that all children are cared for similarly, though who provides care may differ somewhat between families. These null findings are not novel. Previous studies have found negligible associations between individual allomothers, including grandmothers and fathers, and the nutritional status and survival of children in specific contexts (Sear et al. 2000; Vázquez‐Vázquez et al. 2021). What is relatively unusual about this current study is that there are almost no associations seen between care and child outcomes across a wide range of allomothers. previous studies typically find at least one allomother to be associated with improved child outcomes; however, those studies did not directly examine the provision of care. Although we overcome these limitations of previous studies (by measuring caregiving activities instead of using proxy measures of care such as proximity to or absence/presence of kin) our null results lead us to highlight three further considerations in the study of allomaternal care and child health: additive versus substitutive care, confounding by maternal need for support, and limitations of growth measures.

The relationship between allomothering and child outcomes is theoretically predicted to be moderated by maternal investment. Some allomaternal investments are “additive” or in addition to maternal investment, such that they do not reduce the amount of care provided by mothers. Other forms of allomothering are “substitutive” in that they replace maternal care otherwise provided by mothers, allowing mothers to redirect their energy towards other activities (Emmott and Page 2019; Kushnick 2012). Additive care is expected to result in a net benefit, leading to improved child health; much previous research in this area has implicitly assumed that allomothering is additive. Although substitutive care may lead to null or neutral relationships between care provision and health outcomes, and in some cases, may even have detrimental effects on children's health (e.g., if the allomother substituting for the mother is unable to provide the quality of care the mother would have) (Emmott and Mace 2015; Emmott and Page 2019; Kramer and Veile 2018; Page et al. 2021). This is further complicated by the fact that the nature of care (i.e., whether it is additive or substitutive) may vary according to who provides it and the type of care provided and, in certain circumstances, different caregivers might substitute for one another. For example, among Shodagor families in Bangladesh, where fathers provide high levels of childcare, the provision of this care had positive associations with child health, but only when the care being provided was “additive” to maternal care (Starkweather et al. 2021). Ultimately then, we can expect the relationship between allomaternal care and child outcomes to be either positive, negative, or even neutral. Given that we do not have data on the frequency of maternal or allomaternal care, nor on what mothers were doing when allomaternal care was provided, we cannot tell from our data whether allomothering was additive or substitutive. Future data that distinguishes between additive and substitutive types of care, for example, by asking questions about maternal activities while children are looked after by an allomother, is necessary to disentangle this relationship between caregiving and child health further.

Associations between allomothering and child outcomes may also be blurred when caregivers help children who are in greater need, with studies showing that allomaternal support can be responsive to the needs of mothers (Snopkowski and Sear 2015; Schaffnit and Sear 2017). Children who are already unwell may require extra care, leading to allomothers caring for children because they already have, or are at risk of, poorer health. Analyses of childcare and child health using cross‐sectional data may confuse the direction of the association making it appear that specific caregivers are associated with worse child outcomes.

Finally, the lack of associations between caregiving and children's HAZ may also be because HAZ is representative of longer‐term chronic conditions. Thus, an instance of care provision in a short‐term period (2 weeks prior to the survey) may not influence children's HAZ and be better elucidated using longitudinal data. With this logic, however, we would expect some correlation between caregiving and children's WHZ as they both reflect a similar timeframe in the child's life. Given the null results for these associations too, we give more weight to our earlier conclusion that children's caregiving networks appear to neither cause nor buffer children from adverse outcomes, and we do not have sufficient evidence to argue whether or not allomothering is associated with child growth.

We also found that children residing away from their living mothers had similar growth outcomes to children living with their mothers. This is in line with some previous studies which show nonmaternal caregiving to be of similar quality to maternal care. Among the Kipsigis people in Kenya, maternal absence did not diminish the quality of care provided to infants, nor contribute to the infant's distress; and allomothers provided care that equalled quality of maternal care (Borgerhoff Mulder and Milton 1985); and in the Philippines, Agta grandmothers are seen to provide care‐types that are in line with maternal care (Page et al. 2021). Our research elsewhere in Tanzania found that children residing away from living parents had similar growth outcomes to children residing with both parents (Lawson et al. 2017). Also, previous research in the same communities where this study was undertaken shows that children living with both parents were comparable to both orphans and children with one living parent in terms of education (Hedges et al. 2019) and mortality (Urassa et al. 1997). This demonstrates that substitutive or compensatory care is common in the Tanzanian context and emphasizes the local importance of child fostering for at least maintaining children's growth and education, and in some cases preventing extreme outcomes like death (although see Gaydosh 2019 for exception). It also highlights the need to distinguish children whose mothers are absent because of death versus those not residing with their living mothers, as the latter does not appear to be detrimental to children's well‐being in this context—which may differ from contexts where fostering is less common.

We do not put much weight on the few statistically significant associations found between allomothering and child outcomes because we conducted multiple tests, which might represent spurious results because of a Type I error rate inflation (over 56 comparisons the likelihood that one is a Type I error is 95.6% based on an alpha of 0.05). This is further emphasized by the results of the Holm correction for multiple comparisons, which shifts the higher *p* values that previously met the conventional alpha of 0.05 into nonsignificance. As a result, they are likely spurious. Although our results for fathers' caregiving do not hold after the test for multiple corrections, we know from our descriptive results and from other studies that fathers frequently provide significant care to their children both in this context and universally, as is the case for other allomothers. We therefore do not interpret our results as suggesting that paternal care or care from allomothers is unimportant in children's or mothers' lives. Instead, we interpret our full set of analyses as providing little evidence for associations between our measures of allomothering and child height or weight, given point estimates that were mostly close to zero and were inconsistent in significance across models.

### 4.7 Data Limitations and Future Research

It is possible that our measures of care did not capture the complex realities of children's caregiving environments. Our data indicate whether a child had received each type of care at least once from a particular caregiver (e.g., maternal aunt) in the 2 weeks preceding the survey versus not receiving any care from that allomother, and thus we do not know the extent of care provided by that caregiver or whether more than one caregiver in that category had provided care (i.e., two maternal aunts versus one). A measure of frequency, which accounts for all caregivers who provided care and the amount of care provided by each, would be more useful, as receiving care only once may have no impact on growth outcomes, whereas receiving a higher frequency of care may be associated with better growth. In some cases, we may be comparing children who had received care only once versus those who had not received it at all—and as such may not see any real differences in health outcomes. Similarly, having a higher number of allomothers may not correlate with the amount of care received by the child; that is, one child may receive the same amount of care from one allomother that another child receives from seven allomothers; so we do not run analyses using the total number of carers a child had. For future research, collecting data on and examining associations between frequency of care provided to a child by each caregiver and the child's health outcomes may be more instructive than whether the child received care or not, or the number of carers they had. We focused on a few specific types of caregiving in this study because of the nature of the data; asking parents/guardians to report on a wider range of caregiving activities could be explored in the future. It is also plausible that there are pathways other than direct caregiving via which allomothering has a positive impact on children's growth, for example, food sharing and resource provisioning, which can be explored in the future. Another important avenue for future research is to explore whether the combination of care from multiple allomothers has an association with children's outcomes. Analyses of longitudinal data can help show whether care is given to needier (or less healthy) children and clarify the direction of effect between caregiving and worse health outcomes. If longitudinal data are not available or are challenging to collect, then directed qualitative questions that explore why specific caregivers help in different contexts may also elucidate these relationships.

### 4.8 Conclusion

We draw on the strengths of a large sample of children from a rural but urbanizing context to explore associations between children's caregiving environments and growth outcomes. We demonstrate that children received allomothering from a range of individuals, even when mothers are co‐resident. We also show that care from different types of matrilineal kin is positively associated with one another, as is care from fathers and other patrilineal relatives; but that care from fathers tends to be negatively associated with care from matrilineal relatives. However, whereas our results demonstrate minor variations in the relationship between carer, type of care, and children's HAZ and WHZ, we largely see a lack of associations between caregiving and child growth. Our data do not allow us to distinguish between allomothering which is additive to maternal care rather than substitutive, and this may have muted associations between caregiving and child outcomes. Although this research extends beyond the use of proxy measures of care as seen in many previous large‐scale studies on childcare and health, we conclude that even more nuanced measures of children's caregiving environments are needed to help clarify the complicated, but important, link it has with child growth.

## Conflicts of Interest

The authors declare no conflicts of interest.

## Supporting information


Data S1.

## Data Availability

The data that support the findings of this study are available from the corresponding author upon reasonable request.
