# Supplementary material for: Children's Caregiving and Growth in Northwestern Tanzania: Limited Evidence That Support From Specific Caregivers Is Associated With Better Growth
Source: Am J Hum Biol. 2025 Mar 26;37(3):e70029. doi: 10.1002/ajhb.70029 (PMC11947294; doi:10.1002/ajhb.70029)
Supplement: Supplementary file 1 — Data S1. [file AJHB-37-e70029-s001.docx]

**Children’s caregiving and growth in north-western Tanzania: limited evidence that support from specific caregivers is associated with better growth – Supplementary Information**

Anushé Hassan, David W. Lawson, Abigail E. Page, Rebecca Sear, Susan B. Schaffnit and Mark Urassa

**Extended Materials and Methods**

Variables: Height-for-Age and Weight-for-Height Z-scores

A low HAZ refers to “stunting” or “failing to grow” among children below 3 years and “being stunted” or “having failed to grow” among children above 3 years (de Onis and Blössner 2003). This can be a result of long-term malnutrition or inappropriate feeding practices as well as early exposure and recurrent illnesses. HAZ, therefore, describes chronic malnutrition by analysing body length or height in relation to age. A child with a HAZ of less than -2 SD is considered stunted or chronically malnourished, and a child with a HAZ of less than -3 SD is considered severely stunted. A low WHZ refers to wasting or thinness and indicates current or recent extreme weight loss/malnutrition. This can be due to acute starvation and/or severe illness. Usually, low WHZ peaks in the second year of life (de Onis and Blössner 2003). WHZ are used to describe current nutritional status by analysing body weight in relation to body length or height. A child with a WHZ of less than -2 standard deviations (-2SD) from the median of the WHO reference population is considered thin or “wasted” or acutely malnourished, and a child with a WHZ of less than -3 standard deviations (-3 SD) is considered severely wasted.

*Variables: Childcare interview questions*

Table S1 lists the survey questions used during data collection pertaining to the variables utilised in this manuscript.

**Supplementary Table S1 – List of interview questions pertaining to childcare variables (English version)**

| I would now like to ask you some questions about who provides different types of care for (the child). For each type of care please indicate who has provided this care at any time in the past two weeks. You can select more than one person. | |
| --- | --- |
| Type of care | List of allomothers to select |
| Who has washed (the child) at any time in the past two weeks? | 1. Biological mother 2. Biological father 3. Child’s sister/brother 4. Maternal grandparent 5. Paternal grandparent 6. Mother’s sister or brother 7. Father’s sister or brother 8. Stepmother/Stepfather 9. Other |
| Who has provided food or milk for (the child) at any time in the past two weeks? |  |
| Who has played with (the child) at any time in the past two weeks? |  |
| Who has supervised (the child) at any time in the past two weeks? By this we mean, watching (the child) passively or actively to make sure they are safe. |  |
| Who has taken care of (the child) when they were sick or unwell in the past two weeks? |  |

*Analysis: Directed Acyclic Graphs*

We considered a number of child, maternal and household-level variables to include in our models as controls, based on known associations in previous research on childcare and child outcomes. These included: child’s age (continuous in years), child’s sex, if the child was their father’s first child as a proxy for birth order, maternal presence in the household, child’s mother’s age in years, child’s mother’s height, rural/urban residence, number of under 10-year-olds in the household as a measure of competition, reported sickness in the last two weeks, and food insecurity as a proxy for household socio-economic status. ﻿Food insecurity was measured using the Household Food Insecurity (Access) Scale (Coates et al. 2007), which records whether the household experienced problems with accessing food in the past month. Apart from these child, maternal and household-level variables, we also considered including caregiving from each caregiver (the key independent variables described in ‘Data and Methods: Variables’ in the main manuscript) as control variables in each model. We then generated a list of relationships between each of these variables (the considered control variables and the exposure and outcome variables) based on what we expected or knew the direction of effect to be. This initial list was used to generate the first iteration of our DAG (see Figure S1 below for an example DAG testing associations between high-intensity care from fathers and children’s HAZ). The *dagitty* package in R was then used to generate minimally sufficient adjustment sets for each regression model by specifying which of the variables from the list was our outcome variable (e.g., HAZ), and which was our exposure variable (e.g., care from fathers). The minimally sufficient adjustment set for each regression model was the smallest list of variables that should be included as covariates in the specified model (e.g., association between high intensity care from fathers and HAZ). Finally, we checked implied conditional independencies for each DAG: this is a test to see whether the relationships we specified between the variables in our DAG hold within our data, and if there are any relationships between these considered variables in our data that we have not included in our DAG. For example, in our initial list of relationships we had not included a relationship between child’s age and maternal presence in the household, but the implied conditional independencies test showed that this relationship existed in our data and should be included in our DAG. These additional relationships were then included in our DAG and a second iteration of the DAG was produced, and minimally sufficient adjustment sets produced again. This process was repeated till the implied conditional independencies test showed that all relationships between the variables considered for our model had been included in the DAG. Due to potentially varying associations between the covariates and the exposure and outcome variables within each model we generated separate DAGs for each regression model. As such, four DAGs (or list of relationships between variables) were produced: high intensity care and HAZ; high intensity care and WHZ; low intensity care and HAZ; and low intensity care and WHZ. For each of these four DAGs, we generated minimally sufficient adjustment sets separately for the seven different exposure variables (care from each of the seven caregivers). The final iteration of the minimally sufficient adjustment sets produced for each DAG (28 in total) determined which of the initially considered covariates were included in the regression model. Figure S2 below shows the final DAG for the model testing associations between high-intensity care from fathers and children’s HAZ, with variables selected for inclusion in the regression model in grey boxes.

While the minimally sufficient adjustment sets remained consistent across the four broad DAGs (HAZ/WHZ and low/high intensity care), they varied within each depending on which caregiver was under consideration. The adjustment sets indicated that all models contain terms for child’s age, whether the child was their father’s first child, maternal presence in the household, urban/rural residence, number of under 10-year-olds in the household, child’s recent sickness and food insecurity. In addition, the final adjustment sets for care from fathers indicated paternal care models to contain an additional terms for child’s sex, which is in line with previous research using these data that demonstrated paternal care to be sex-dependent (Hassan et al. 2019). Adjustment sets for maternal and paternal grandparental care indicated these models should include a control for care from fathers. Adjustment sets for maternal and paternal aunts/uncles showed that these models should control for care from maternal and paternal grandparents, respectively. From the original list of relationships between variables (the original DAG) a separate minimally sufficient adjustment set was created for the model exploring if maternal presence predicted child outcomes. This showed that only child’s age and whether the child was their father’s first child should be retained in the regression models for both child outcomes (HAZ and WHZ).

**Supplementary Table S2 – List of variables used in the Directed Acyclic Graphs**

| outcome | The outcome variable, either HAZ or WHZ depending on the model |
| --- | --- |
| fath | Whether or not the child received care (high-intensity or low-intensity, depending on the model) from their father in the two weeks preceding the survey (in Figures S1 and S2, ‘fath’ is the exposure variable) |
| sex | The child’s sex (female or male) |
| age | The child’s age in decimal years |
| birthorder | Whether or not the focal child was the first born child of their father |
| sibcount | The number of under 10-year old children living the same household as the focal child |
| sick | Whether or not the child had been sick in the two weeks preceding the survey |
| fs | Household food security level (proxy for SES) |
| urban | Whether the child’s household was in a rural or urban setting |
| matpres | Whether the child’s biological mother resided in the same household as the child or resided apart from the child (excluding dead mothers) |
| sib | Whether or not the child received care (high-intensity or low-intensity, depending on the model) from their sibling(s) in the two weeks preceding the survey |
| MGP | Whether or not the child received care (high-intensity or low-intensity, depending on the model) from their maternal grandparents in the two weeks preceding the survey |
| PGP | Whether or not the child received care (high-intensity or low-intensity, depending on the model) from their paternal grandparents in the two weeks preceding the survey |
| Mkin | Whether or not the child received care (high-intensity or low-intensity, depending on the model) from their maternal aunts/uncles in the two weeks preceding the survey |
| Pkin | Whether or not the child received care (high-intensity or low-intensity, depending on the model) from their paternal aunts/uncles in the two weeks preceding the survey |
| nonkin | Whether or not the child received care (high-intensity or low-intensity, depending on the model) from their distant kin / non-kin in the two weeks preceding the survey |

**Supplementary Figure S1 –** Directed Acyclic Graph illustrating causal relationships between variables for the model testing associations between high intensity care from fathers (exposure, green with triangle) and children’s HAZ (outcome, blue with straight line). See Table S2 for details on the list of variables included in this DAG.


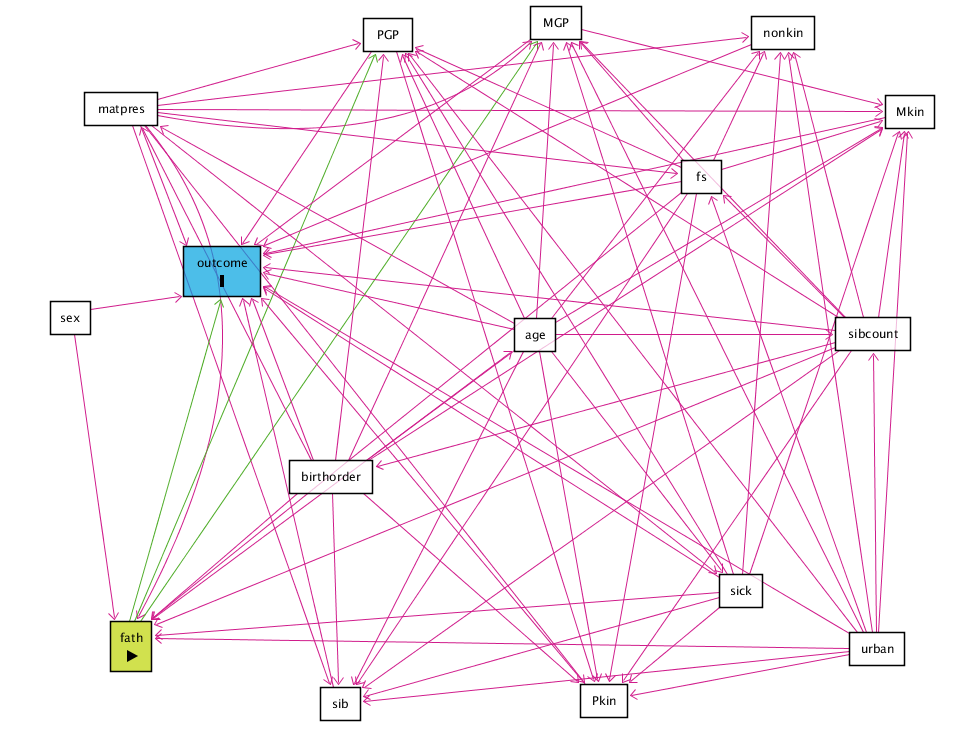


**Supplementary Figure 2 –** Directed Acyclic Graph illustrating causal relationships between variables for the model testing associations between high intensity care from fathers (‘fath’ - exposure, green with triangle) and children’s HAZ (‘outcome’ - outcome, blue with straight line), with variables selected for inclusion in the regression model (confounders) in grey.

**
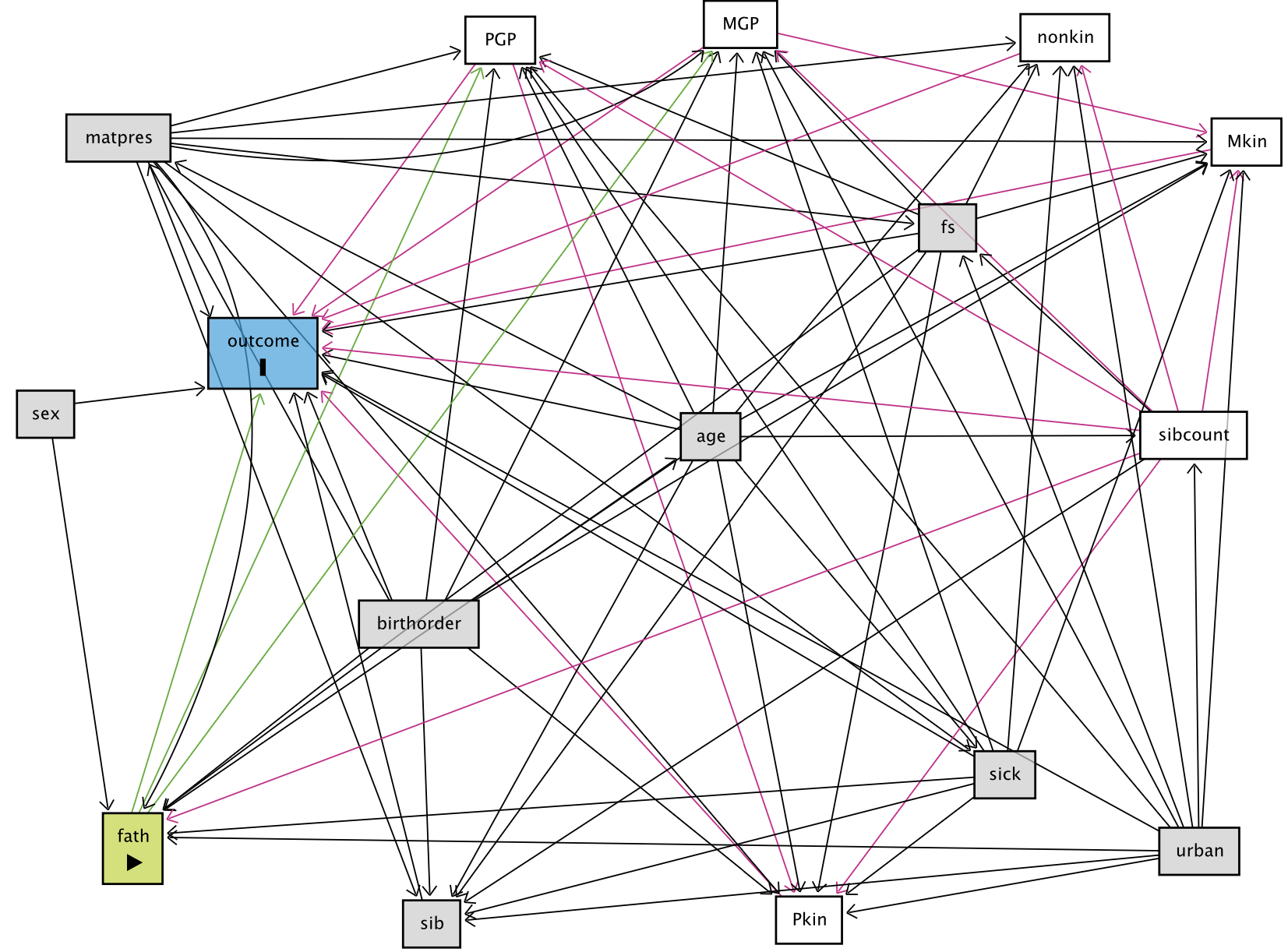
**

**Extended Results**

*Children’s HAZ and WHZ distribution*

Supplementary Figure S3 shows the range of HAZ and WHZ scores in our sample along with the WHO cut-offs for ‘stunting’ and ‘wasting’.


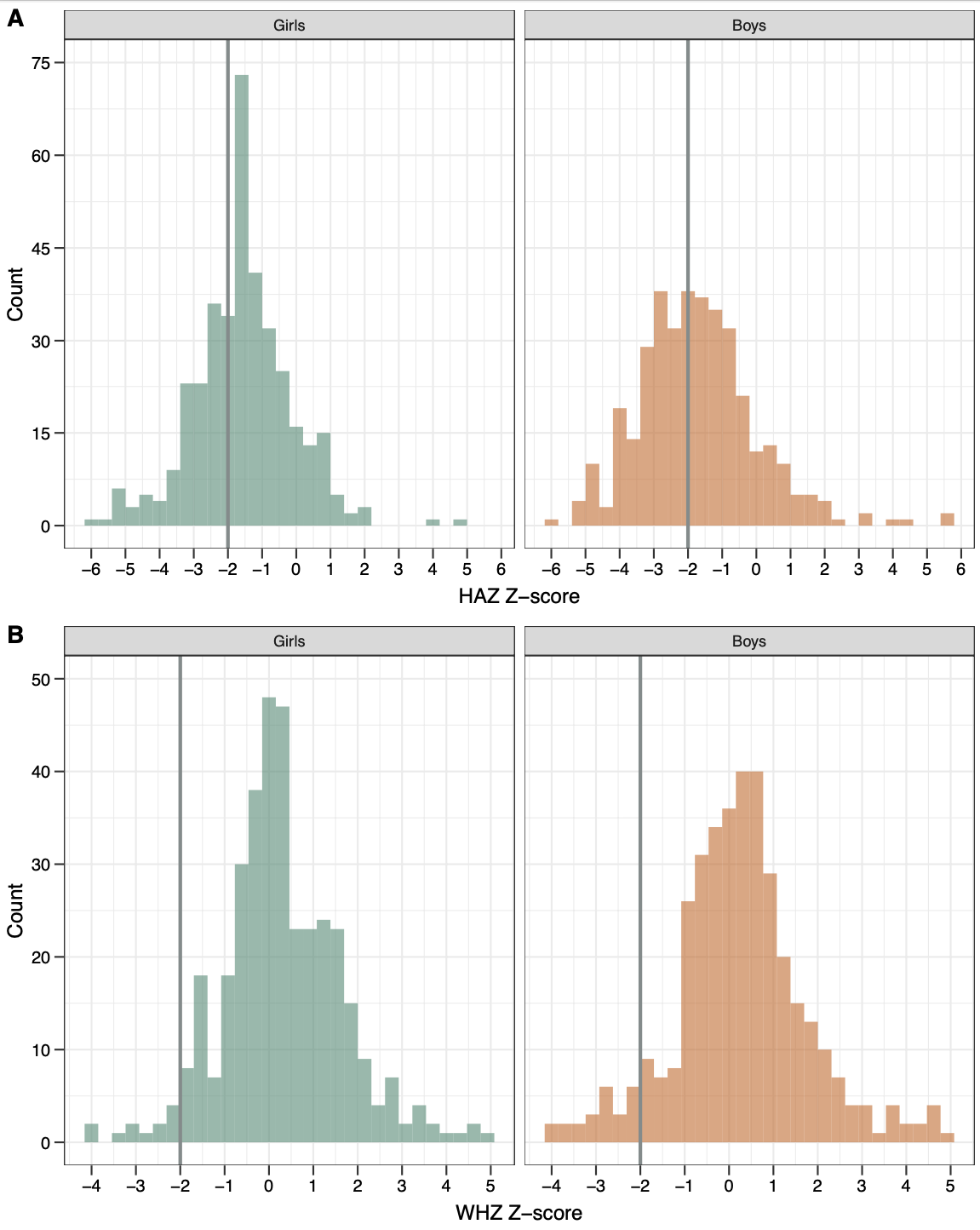


**Figure S3 -** Histograms of HAZ and WHZ by child’s sex. Girls are represented by green bars, boys by orange bars. The solid grey line represents the WHO cut-offs for stunting (HAZ) and wasting (WHZ).

*Model results*

**Supplementary Table S3 – Maternal presence and child growth outcomes.** The row ‘mother co-resident’ shows the effect of having a co-resident mother on children’s HAZ and WHZ controlling for child age and if the child was their father’s first child.

|  | HAZ | | | | WHZ | | | |
| --- | --- | --- | --- | --- | --- | --- | --- | --- |
|  | **Estimate** | **95% CI** | | **p-value** | **Estimate** | **95% CI** | | **p-value** |
| (Intercept) | -1.43 | -1.68 | -1.18 | <2e-16 | 0.52 | 0.30 | 0.75 | 0.00 |
| Mother co-resident (ref: non-res) | -0.11 | -0.55 | 0.33 | 0.62 | -0.20 | -0.59 | 0.19 | 0.32 |
| Child's age (in years) | -0.06 | -0.15 | 0.03 | 0.20 | -0.11 | -0.19 | -0.03 | 0.01 |
| Child is first child (ref: not first child) | -0.30 | -0.59 | -0.01 | 0.04 | 0.28 | 0.03 | 0.54 | 0.03 |

**Supplementary Table S4 – Care from fathers and children’s growth outcomes.** The row ‘care received and mother absent’ shows the effect of receiving high/low care from fathers on children’s HAZ/WHZ for children who did not live with their mothers. The row ‘care received and mother present’ shows the effect of receiving high/low care from fathers on children’s HAZ/WHZ for children who lived with their mothers. The rows ‘child’s age’ till ‘urban residence’ are control variables. pH = pHolm.

|  |  | HAZ | | | | | | | |  |  | WHZ | | | | | | | |  |
| --- | --- | --- | --- | --- | --- | --- | --- | --- | --- | --- | --- | --- | --- | --- | --- | --- | --- | --- | --- | --- |
| Caregiver: fathers | **High-intensity care** | | | |  | **Low-intensity care** | | | |  | **High-intensity care** | | | |  | **Low-intensity care** | | | |  |
| Mother is non-resident | **est.** | **95% CI** | | **p** | **pH** | **est.** | **95% CI** | | **p** | **pH** | **est.** | **95% CI** | | **p** | **pH** | **est.** | **95% CI** | | **p** | **pH** |
| (Intercept) | -1.32 | -1.98 | -0.66 | 0.00 |  | -1.17 | -1.85 | -0.49 | 0.00 |  | 0.48 | -0.11 | 1.07 | 0.11 |  | 0.46 | -0.14 | 1.07 | 0.14 |  |
| Care received | **1.75** | **0.43** | **3.08** | **0.01** | 0.07 | -0.08 | -1.05 | 0.90 | 0.87 | 1.00 | 0.21 | -0.97 | 1.39 | 0.73 | 1.00 | 0.39 | -0.47 | 1.26 | 0.37 | 1.00 |
| Mother present | 0.29 | -0.18 | 0.76 | 0.22 |  | 0.06 | -0.46 | 0.57 | 0.83 |  | 0.14 | -0.28 | 0.55 | 0.52 |  | 0.26 | -0.20 | 0.71 | 0.27 |  |
| Child’s age | -0.07 | -0.16 | 0.02 | 0.12 |  | -0.07 | -0.16 | 0.02 | 0.13 |  | -0.12 | -0.20 | -0.04 | 0.00 |  | -0.13 | -0.21 | -0.05 | 0.00 |  |
| Food security | -0.01 | -0.03 | 0.00 | 0.07 |  | -0.01 | -0.03 | 0.00 | 0.08 |  | 0.00 | -0.01 | 0.02 | 0.86 |  | 0.00 | -0.01 | 0.01 | 0.96 |  |
| Child is boy (ref: girl) | -0.21 | -0.44 | 0.03 | 0.08 |  | -0.19 | -0.42 | 0.05 | 0.11 |  | -0.05 | -0.26 | 0.16 | 0.63 |  | -0.04 | -0.24 | 0.17 | 0.73 |  |
| Number under-10s in house | -0.03 | -0.09 | 0.03 | 0.34 |  | -0.03 | -0.09 | 0.04 | 0.41 |  | -0.03 | -0.08 | 0.03 | 0.34 |  | -0.03 | -0.09 | 0.03 | 0.29 |  |
| Child was sick (ref: not sick) | 0.05 | -0.22 | 0.31 | 0.71 |  | 0.03 | -0.24 | 0.29 | 0.83 |  | -0.30 | -0.53 | -0.06 | 0.01 |  | -0.26 | -0.50 | -0.03 | 0.03 |  |
| Child is first child (ref: not first child) | -0.34 | -0.63 | -0.05 | 0.02 |  | -0.33 | -0.62 | -0.03 | 0.03 |  | 0.29 | 0.04 | 0.55 | 0.03 |  | 0.29 | 0.02 | 0.55 | 0.03 |  |
| Urban residence (ref: rural) | 0.10 | -0.14 | 0.33 | 0.43 |  | 0.11 | -0.14 | 0.35 | 0.39 |  | -0.04 | -0.25 | 0.18 | 0.74 |  | -0.04 | -0.26 | 0.18 | 0.72 |  |
| Received care:mother present | **-1.86** | **-3.21** | **-0.52** | **0.01** |  | 0.07 | -0.94 | 1.08 | 0.89 |  | 0.07 | -1.13 | 1.27 | 0.91 |  | -0.30 | -1.19 | 0.60 | 0.52 |  |
|  |  |  |  |  |  |  |  |  |  |  |  |  |  |  |  |  |  |  |  |  |
| Mother is co-resident | **est.** | **95% CI** | | **p** | **pH** | **est.** | **95% CI** | | **p** | **pH** | **est.** | **95% CI** | | **p** | **pH** | **est.** | **95% CI** | | **p** | **pH** |
| (Intercept) | -1.03 | -1.50 | -0.55 | 0.00 |  | -1.11 | -1.61 | -0.62 | 0.00 |  | 0.62 | 0.19 | 1.04 | 0.00 |  | 0.72 | 0.28 | 1.16 | 0.00 |  |
| Care received | -0.11 | -0.36 | 0.13 | 0.37 | 1.00 | -0.01 | -0.27 | 0.26 | 0.97 | 1.00 | **0.28** | **0.06** | **0.50** | **0.01** | 0.07 | 0.10 | -0.14 | 0.33 | 0.41 | 0.82 |
| Mother absent | -0.29 | -0.76 | 0.18 | 0.22 |  | -0.06 | -0.57 | 0.46 | 0.83 |  | -0.14 | -0.55 | 0.28 | 0.52 |  | -0.26 | -0.71 | 0.20 | 0.27 |  |
| Child’s age | -0.07 | -0.16 | 0.02 | 0.12 |  | -0.07 | -0.16 | 0.02 | 0.13 |  | -0.12 | -0.20 | -0.04 | 0.00 |  | -0.13 | -0.21 | -0.05 | 0.00 |  |
| Food security | -0.01 | -0.03 | 0.00 | 0.07 |  | -0.01 | -0.03 | 0.00 | 0.08 |  | 0.00 | -0.01 | 0.02 | 0.86 |  | 0.00 | -0.01 | 0.01 | 0.96 |  |
| Child is boy (ref: girl) | -0.21 | -0.44 | 0.03 | 0.08 |  | -0.19 | -0.42 | 0.05 | 0.11 |  | -0.05 | -0.26 | 0.16 | 0.63 |  | -0.04 | -0.24 | 0.17 | 0.73 |  |
| Number under-10s in house | -0.03 | -0.09 | 0.03 | 0.34 |  | -0.03 | -0.09 | 0.04 | 0.41 |  | -0.03 | -0.08 | 0.03 | 0.34 |  | -0.03 | -0.09 | 0.03 | 0.29 |  |
| Child was sick (ref: not sick) | 0.05 | -0.22 | 0.31 | 0.71 |  | 0.03 | -0.24 | 0.29 | 0.83 |  | -0.30 | -0.53 | -0.06 | 0.01 |  | -0.26 | -0.50 | -0.03 | 0.03 |  |
| Child is first child (ref: not first child) | -0.34 | -0.63 | -0.05 | 0.02 |  | -0.33 | -0.62 | -0.03 | 0.03 |  | 0.29 | 0.04 | 0.55 | 0.03 |  | 0.29 | 0.02 | 0.55 | 0.03 |  |
| Urban residence (ref: rural) | 0.10 | -0.14 | 0.33 | 0.43 |  | 0.11 | -0.14 | 0.35 | 0.39 |  | -0.04 | -0.25 | 0.18 | 0.74 |  | -0.04 | -0.26 | 0.18 | 0.72 |  |
| Received care:mother absent | **1.86** | **0.52** | **3.21** | **0.01** |  | -0.07 | -1.08 | 0.94 | 0.89 |  | -0.07 | -1.27 | 1.13 | 0.91 |  | 0.30 | -0.60 | 1.19 | 0.52 |  |

**Supplementary Table S5 – Care from siblings and children’s growth outcomes.** The row ‘care received and mother absent’ shows the effect of receiving high/low care from siblings on children’s HAZ/WHZ for children who did not live with their mothers. The row ‘care received and mother present’ shows the effect of receiving high/low care from siblings on children’s HAZ/WHZ for children who lived with their mothers. The rows ‘child’s age’ till ‘urban residence’ are control variables. pH = pHolm.

|  |  | HAZ | | | | | | | |  |  | WHZ | | | | | | | |  |
| --- | --- | --- | --- | --- | --- | --- | --- | --- | --- | --- | --- | --- | --- | --- | --- | --- | --- | --- | --- | --- |
| Caregiver: siblings | **High-intensity care** | | | |  | **Low-intensity care** | | | |  | **High-intensity care** | | | |  | **Low-intensity care** | | | |  |
| Mother is non-resident | **est.** | **95% CI** | | **p** | **pH** | **est.** | **95% CI** | | **p** | **pH** | **est.** | **95% CI** | | **p** | **pH** | **est.** | **95% CI** | | **p** | **pH** |
| (Intercept) | -1.65 | -2.60 | -0.71 | 0.00 |  | -1.30 | -1.97 | -0.63 | 0.00 |  | 0.10 | -0.74 | 0.93 | 0.82 |  | 0.44 | -0.15 | 1.04 | 0.14 |  |
| Care received | 0.49 | -0.44 | 1.42 | 0.30 | 1.00 | 0.01 | -0.97 | 0.98 | 0.99 | 1.00 | 0.58 | -0.24 | 1.41 | 0.17 | 1.00 | 0.49 | -0.37 | 1.35 | 0.27 | 1.00 |
| Mother present | 0.35 | -0.51 | 1.20 | 0.43 |  | 0.09 | -0.41 | 0.59 | 0.72 |  | **0.83** | **0.07** | **1.59** | **0.03** |  | 0.27 | -0.17 | 0.72 | 0.22 |  |
| Child’s age | -0.08 | -0.17 | 0.01 | 0.10 |  | -0.07 | -0.16 | 0.02 | 0.13 |  | -0.11 | -0.20 | -0.03 | 0.01 |  | -0.13 | -0.21 | -0.05 | 0.00 |  |
| Food security | -0.01 | -0.03 | 0.00 | 0.08 |  | -0.01 | -0.03 | 0.00 | 0.07 |  | 0.00 | -0.01 | 0.01 | 0.95 |  | 0.00 | -0.01 | 0.01 | 0.93 |  |
| Number under-10s in house | -0.03 | -0.09 | 0.03 | 0.37 |  | -0.02 | -0.09 | 0.04 | 0.44 |  | -0.03 | -0.08 | 0.03 | 0.31 |  | -0.03 | -0.09 | 0.02 | 0.24 |  |
| Child was sick (ref: not sick) | 0.02 | -0.24 | 0.29 | 0.87 |  | 0.03 | -0.24 | 0.29 | 0.84 |  | -0.26 | -0.50 | -0.03 | 0.03 |  | -0.28 | -0.51 | -0.04 | 0.02 |  |
| Child's is first child (ref: not first child) | -0.26 | -0.57 | 0.05 | 0.10 |  | -0.32 | -0.62 | -0.02 | 0.04 |  | 0.22 | -0.06 | 0.49 | 0.12 |  | 0.32 | 0.05 | 0.58 | 0.02 |  |
| Urban residence (ref: rural) | 0.12 | -0.12 | 0.36 | 0.34 |  | 0.11 | -0.13 | 0.35 | 0.38 |  | -0.07 | -0.28 | 0.14 | 0.52 |  | -0.06 | -0.27 | 0.16 | 0.60 |  |
| Received care:mother present | -0.35 | -1.33 | 0.63 | 0.48 |  | -0.01 | -1.02 | 1.00 | 0.98 |  | -0.79 | -1.66 | 0.08 | 0.07 |  | -0.29 | -1.19 | 0.61 | 0.53 |  |
|  |  |  |  |  |  |  |  |  |  |  |  |  |  |  |  |  |  |  |  |  |
| Mother is co-resident | **est.** | **95% CI** | | **p** | **pH** | **est.** | **95% CI** | | **p** | **pH** | **est.** | **95% CI** | | **p** | **pH** | **est.** | **95% CI** | | **p** | **pH** |
| (Intercept) | -1.31 | -1.79 | -0.82 | 0.00 |  | -1.21 | -1.65 | -0.77 | 0.00 |  | 0.92 | 0.49 | 1.36 | 0.00 |  | 0.72 | 0.33 | 1.11 | 0.00 |  |
| Care received | 0.14 | -0.20 | 0.47 | 0.41 | 1.00 | 0.00 | -0.26 | 0.25 | 0.97 | 1.00 | -0.21 | -0.51 | 0.09 | 0.17 | 1.00 | 0.20 | -0.03 | 0.43 | 0.09 | 0.63 |
| Mother absent | -0.35 | -1.20 | 0.51 | 0.43 |  | -0.09 | -0.59 | 0.41 | 0.72 |  | **-0.83** | **-1.59** | **-0.07** | **0.03** |  | -0.27 | -0.72 | 0.17 | 0.22 |  |
| Child’s age | -0.08 | -0.17 | 0.01 | 0.10 |  | -0.07 | -0.16 | 0.02 | 0.13 |  | -0.11 | -0.20 | -0.03 | 0.01 |  | -0.13 | -0.21 | -0.05 | 0.00 |  |
| Food security | -0.01 | -0.03 | 0.00 | 0.08 |  | -0.01 | -0.03 | 0.00 | 0.07 |  | 0.00 | -0.01 | 0.01 | 0.95 |  | 0.00 | -0.01 | 0.01 | 0.93 |  |
| Number under-10s in house | -0.03 | -0.09 | 0.03 | 0.37 |  | -0.02 | -0.09 | 0.04 | 0.44 |  | -0.03 | -0.08 | 0.03 | 0.31 |  | -0.03 | -0.09 | 0.02 | 0.24 |  |
| Child was sick (ref: not sick) | 0.02 | -0.24 | 0.29 | 0.87 |  | 0.03 | -0.24 | 0.29 | 0.84 |  | -0.26 | -0.50 | -0.03 | 0.03 |  | -0.28 | -0.51 | -0.04 | 0.02 |  |
| Child's is first child (ref: not first child) | -0.26 | -0.57 | 0.05 | 0.10 |  | -0.32 | -0.62 | -0.02 | 0.04 |  | 0.22 | -0.06 | 0.49 | 0.12 |  | 0.32 | 0.05 | 0.58 | 0.02 |  |
| Urban residence (ref: rural) | 0.12 | -0.12 | 0.36 | 0.34 |  | 0.11 | -0.13 | 0.35 | 0.38 |  | -0.07 | -0.28 | 0.14 | 0.52 |  | -0.06 | -0.27 | 0.16 | 0.60 |  |
| Received care:mother absent | 0.35 | -0.63 | 1.33 | 0.48 |  | 0.01 | -1.00 | 1.02 | 0.98 |  | *0.79* | *-0.08* | *1.66* | *0.07* |  | 0.29 | -0.61 | 1.19 | 0.53 |  |

**Supplementary Table S6 – Care from maternal grandparents and children’s growth outcomes.** The row ‘care received and mother absent’ shows the effect of receiving high/low care from maternal grandparents on children’s HAZ/WHZ for children who did not live with their mothers. The row ‘care received and mother present’ shows the effect of receiving high/low care from maternal grandparents on children’s HAZ/WHZ for children who lived with their mothers. The rows ‘child’s age’ till ‘received high care from father’ are control variables. pH = pHolm.

|  |  | HAZ | | | | | | | |  |  | WHZ | | | | | | | |  |
| --- | --- | --- | --- | --- | --- | --- | --- | --- | --- | --- | --- | --- | --- | --- | --- | --- | --- | --- | --- | --- |
| Caregiver: maternal grandparents | **High-intensity care** | | | |  | **Low-intensity care** | | | |  | **High-intensity care** | | | |  | **Low-intensity care** | | | |  |
| Mother is non-resident | **est.** | **95% CI** | | **p** | **pH** | **est.** | **95% CI** | | **p** | **pH** | **est.** | **95% CI** | | **p** | **pH** | **est.** | **95% CI** | | **p** | **pH** |
| (Intercept) | -1.28 | -2.05 | -0.51 | 0.00 |  | -1.21 | -1.98 | -0.44 | 0.00 |  | 0.44 | -0.25 | 1.13 | 0.21 |  | 0.55 | -0.13 | 1.24 | 0.11 |  |
| Care received | -0.02 | -0.83 | 0.79 | 0.96 | 1.00 | -0.19 | -1.01 | 0.63 | 0.65 | 1.00 | 0.02 | -0.71 | 0.74 | 0.96 | 1.00 | -0.11 | -0.85 | 0.62 | 0.76 | 1.00 |
| Mother present | 0.18 | -0.45 | 0.81 | 0.58 |  | 0.14 | -0.47 | 0.75 | 0.65 |  | 0.15 | -0.41 | 0.71 | 0.60 |  | 0.16 | -0.38 | 0.71 | 0.56 |  |
| Child’s age | -0.08 | -0.17 | 0.01 | 0.08 |  | -0.07 | -0.16 | 0.02 | 0.13 |  | -0.11 | -0.20 | -0.03 | 0.00 |  | -0.13 | -0.21 | -0.05 | 0.00 |  |
| Child's is first child (ref: not first child) | -0.26 | -0.55 | 0.04 | 0.09 |  | -0.26 | -0.56 | 0.04 | 0.09 |  | 0.28 | 0.02 | 0.55 | 0.04 |  | 0.31 | 0.04 | 0.58 | 0.02 |  |
| Food security | -0.01 | -0.03 | 0.00 | 0.08 |  | -0.01 | -0.03 | 0.00 | 0.09 |  | 0.00 | -0.01 | 0.02 | 0.90 |  | 0.00 | -0.01 | 0.01 | 0.96 |  |
| Child was sick (ref: not sick) | 0.04 | -0.23 | 0.31 | 0.76 |  | 0.04 | -0.23 | 0.30 | 0.79 |  | -0.30 | -0.53 | -0.06 | 0.01 |  | -0.26 | -0.50 | -0.03 | 0.03 |  |
| Number under-10s in house | -0.02 | -0.08 | 0.04 | 0.49 |  | -0.02 | -0.08 | 0.04 | 0.50 |  | -0.03 | -0.08 | 0.03 | 0.33 |  | -0.03 | -0.08 | 0.03 | 0.32 |  |
| Urban residence (ref: rural) | 0.10 | -0.14 | 0.34 | 0.41 |  | 0.09 | -0.15 | 0.34 | 0.45 |  | -0.04 | -0.25 | 0.18 | 0.74 |  | -0.04 | -0.26 | 0.18 | 0.71 |  |
| Received high care from father (ref: no) | -0.11 | -0.35 | 0.14 | 0.39 |  | -0.16 | -0.43 | 0.12 | 0.27 |  | 0.28 | 0.06 | 0.49 | 0.01 |  | 0.08 | -0.16 | 0.33 | 0.50 |  |
| Received care:mother present | -0.24 | -1.12 | 0.63 | 0.58 |  | -0.18 | -1.06 | 0.69 | 0.68 |  | 0.02 | -0.76 | 0.79 | 0.96 |  | 0.03 | -0.75 | 0.81 | 0.93 |  |
|  |  |  |  |  |  |  |  |  |  |  |  |  |  |  |  |  |  |  |  |  |
| Mother is co-resident | **est.** | **95% CI** | | **p** | **pH** | **est.** | **95% CI** | | **p** | **pH** | **est.** | **95% CI** | | **p** | **pH** | **est.** | **95% CI** | | **p** | **pH** |
| (Intercept) | -1.10 | -1.57 | -0.63 | 0.00 |  | -1.07 | -1.56 | -0.57 | 0.00 |  | 0.59 | 0.17 | 1.01 | 0.01 |  | 0.72 | 0.28 | 1.16 | 0.00 |  |
| Care received | -0.26 | -0.57 | 0.04 | 0.09 | 0.54 | **-0.37** | **-0.71** | **-0.03** | **0.03** | 0.21 | 0.03 | -0.24 | 0.31 | 0.80 | 1.00 | -0.08 | -0.39 | 0.23 | 0.61 | 0.82 |
| Mother absent | -0.18 | -0.81 | 0.45 | 0.58 |  | -0.14 | -0.75 | 0.47 | 0.65 |  | -0.15 | -0.71 | 0.41 | 0.60 |  | -0.16 | -0.71 | 0.38 | 0.56 |  |
| Child’s age | -0.08 | -0.17 | 0.01 | 0.08 |  | -0.07 | -0.16 | 0.02 | 0.13 |  | -0.11 | -0.20 | -0.03 | 0.00 |  | -0.13 | -0.21 | -0.05 | 0.00 |  |
| Child's is first child (ref: not first child) | -0.26 | -0.55 | 0.04 | 0.09 |  | -0.26 | -0.56 | 0.04 | 0.09 |  | 0.28 | 0.02 | 0.55 | 0.04 |  | 0.31 | 0.04 | 0.58 | 0.02 |  |
| Food security | -0.01 | -0.03 | 0.00 | 0.08 |  | -0.01 | -0.03 | 0.00 | 0.09 |  | 0.00 | -0.01 | 0.02 | 0.90 |  | 0.00 | -0.01 | 0.01 | 0.96 |  |
| Child was sick (ref: not sick) | 0.04 | -0.23 | 0.31 | 0.76 |  | 0.04 | -0.23 | 0.30 | 0.79 |  | -0.30 | -0.53 | -0.06 | 0.01 |  | -0.26 | -0.50 | -0.03 | 0.03 |  |
| Number under-10s in house | -0.02 | -0.08 | 0.04 | 0.49 |  | -0.02 | -0.08 | 0.04 | 0.50 |  | -0.03 | -0.08 | 0.03 | 0.33 |  | -0.03 | -0.08 | 0.03 | 0.32 |  |
| Urban residence (ref: rural) | 0.10 | -0.14 | 0.34 | 0.41 |  | 0.09 | -0.15 | 0.34 | 0.45 |  | -0.04 | -0.25 | 0.18 | 0.74 |  | -0.04 | -0.26 | 0.18 | 0.71 |  |
| Received high care from father (ref: no) | -0.11 | -0.35 | 0.14 | 0.39 |  | -0.16 | -0.43 | 0.12 | 0.27 |  | 0.28 | 0.06 | 0.49 | 0.01 |  | 0.08 | -0.16 | 0.33 | 0.50 |  |
| Received care:mother absent | 0.24 | -0.63 | 1.12 | 0.58 |  | 0.18 | -0.69 | 1.06 | 0.68 |  | -0.02 | -0.79 | 0.76 | 0.96 |  | -0.03 | -0.81 | 0.75 | 0.93 |  |

**Supplementary Table S7 – Care from paternal grandparents and children’s growth outcomes.** The row ‘care received and mother absent’ shows the effect of receiving high/low care from paternal grandparents on children’s HAZ/WHZ for children who did not live with their mothers. The row ‘care received and mother present’ shows the effect of receiving high/low care from paternal grandparents on children’s HAZ/WHZ for children who lived with their mothers. The rows ‘child’s age’ till ‘received high care from father’ are control variables. pH = pHolm.

|  |  | HAZ | | | | | | | |  |  | WHZ | | | | | | | |  |
| --- | --- | --- | --- | --- | --- | --- | --- | --- | --- | --- | --- | --- | --- | --- | --- | --- | --- | --- | --- | --- |
| Caregiver: paternal grandparents | **High-intensity care** | | | |  | **Low-intensity care** | | | |  | **High-intensity care** | | | |  | **Low-intensity care** | | | |  |
| Mother is non-resident | **est.** | **95% CI** | | **p** | **pH** | **est.** | **95% CI** | | **p** | **pH** | **est.** | **95% CI** | | **p** | **pH** | **est.** | **95% CI** | | **p** | **pH** |
| (Intercept) | -1.16 | -1.88 | -0.44 | 0.00 |  | -1.25 | -1.96 | -0.54 | 0.00 |  | 0.35 | -0.29 | 0.99 | 0.28 |  | 0.49 | -0.14 | 1.13 | 0.13 |  |
| Care received | -0.27 | -1.10 | 0.57 | 0.53 | 1.00 | -0.09 | -0.93 | 0.76 | 0.84 | 1.00 | 0.20 | -0.53 | 0.94 | 0.59 | 1.00 | 0.13 | -0.62 | 0.88 | 0.73 | 1.00 |
| Mother present | 0.00 | -0.57 | 0.56 | 0.99 |  | 0.06 | -0.51 | 0.63 | 0.83 |  | 0.25 | -0.25 | 0.75 | 0.33 |  | 0.22 | -0.29 | 0.72 | 0.40 |  |
| Child’s age | -0.07 | -0.16 | 0.02 | 0.12 |  | -0.07 | -0.16 | 0.02 | 0.14 |  | -0.12 | -0.20 | -0.04 | 0.00 |  | -0.12 | -0.20 | -0.04 | 0.00 |  |
| Child's is first child (ref: not first child) | -0.32 | -0.61 | -0.02 | 0.03 |  | -0.32 | -0.62 | -0.02 | 0.03 |  | 0.30 | 0.04 | 0.56 | 0.02 |  | 0.26 | 0.00 | 0.53 | 0.05 |  |
| Food security | -0.02 | -0.03 | 0.00 | 0.06 |  | -0.02 | -0.03 | 0.00 | 0.07 |  | 0.00 | -0.01 | 0.02 | 0.89 |  | 0.00 | -0.01 | 0.01 | 0.94 |  |
| Child was sick (ref: not sick) | 0.03 | -0.24 | 0.30 | 0.83 |  | 0.02 | -0.24 | 0.29 | 0.85 |  | -0.29 | -0.53 | -0.05 | 0.02 |  | -0.27 | -0.51 | -0.04 | 0.02 |  |
| Number under-10s in house | -0.03 | -0.09 | 0.04 | 0.43 |  | -0.03 | -0.09 | 0.04 | 0.44 |  | -0.02 | -0.08 | 0.03 | 0.41 |  | -0.04 | -0.09 | 0.02 | 0.19 |  |
| Urban residence (ref: rural) | 0.10 | -0.14 | 0.34 | 0.41 |  | 0.10 | -0.14 | 0.35 | 0.41 |  | -0.04 | -0.26 | 0.17 | 0.70 |  | -0.03 | -0.24 | 0.19 | 0.81 |  |
| Received high care from father (ref: no) | -0.07 | -0.32 | 0.18 | 0.58 |  | -0.03 | -0.29 | 0.23 | 0.82 |  | 0.28 | 0.06 | 0.50 | 0.01 |  | 0.08 | -0.15 | 0.31 | 0.49 |  |
| Received care:mother present | 0.30 | -0.60 | 1.19 | 0.51 |  | 0.10 | -0.81 | 1.02 | 0.82 |  | -0.31 | -1.10 | 0.49 | 0.45 |  | 0.14 | -0.67 | 0.95 | 0.74 |  |
|  |  |  |  |  |  |  |  |  |  |  |  |  |  |  |  |  |  |  |  |  |
| Mother is co-resident | **est.** | **95% CI** | | **p** | **pH** | **est.** | **95% CI** | | **p** | **pH** | **est.** | **95% CI** | | **p** | **pH** | **est.** | **95% CI** | | **p** | **pH** |
| (Intercept) | -1.17 | -1.63 | -0.70 | 0.00 |  | -1.19 | -1.67 | -0.70 | 0.00 |  | 0.60 | 0.18 | 1.01 | 0.00 |  | 0.71 | 0.28 | 1.14 | 0.00 |  |
| Care received | 0.03 | -0.34 | 0.40 | 0.87 | 1.00 | 0.01 | -0.38 | 0.41 | 0.94 | 1.00 | -0.10 | -0.43 | 0.23 | 0.55 | 1.00 | 0.27 | -0.08 | 0.62 | 0.13 | 0.72 |
| Mother absent | 0.00 | -0.56 | 0.57 | 0.99 |  | -0.06 | -0.63 | 0.51 | 0.83 |  | -0.25 | -0.75 | 0.25 | 0.33 |  | -0.22 | -0.72 | 0.29 | 0.40 |  |
| Child’s age | -0.07 | -0.16 | 0.02 | 0.12 |  | -0.07 | -0.16 | 0.02 | 0.14 |  | -0.12 | -0.20 | -0.04 | 0.00 |  | -0.12 | -0.20 | -0.04 | 0.00 |  |
| Child's is first child (ref: not first child) | -0.32 | -0.61 | -0.02 | 0.03 |  | -0.32 | -0.62 | -0.02 | 0.03 |  | 0.30 | 0.04 | 0.56 | 0.02 |  | 0.26 | 0.00 | 0.53 | 0.05 |  |
| Food security | -0.02 | -0.03 | 0.00 | 0.06 |  | -0.02 | -0.03 | 0.00 | 0.07 |  | 0.00 | -0.01 | 0.02 | 0.89 |  | 0.00 | -0.01 | 0.01 | 0.94 |  |
| Child was sick (ref: not sick) | 0.03 | -0.24 | 0.30 | 0.83 |  | 0.02 | -0.24 | 0.29 | 0.85 |  | -0.29 | -0.53 | -0.05 | 0.02 |  | -0.27 | -0.51 | -0.04 | 0.02 |  |
| Number under-10s in house | -0.03 | -0.09 | 0.04 | 0.43 |  | -0.03 | -0.09 | 0.04 | 0.44 |  | -0.02 | -0.08 | 0.03 | 0.41 |  | -0.04 | -0.09 | 0.02 | 0.19 |  |
| Urban residence (ref: rural) | 0.10 | -0.14 | 0.34 | 0.41 |  | 0.10 | -0.14 | 0.35 | 0.41 |  | -0.04 | -0.26 | 0.17 | 0.70 |  | -0.03 | -0.24 | 0.19 | 0.81 |  |
| Received high care from father (ref: no) | -0.07 | -0.32 | 0.18 | 0.58 |  | -0.03 | -0.29 | 0.23 | 0.82 |  | 0.28 | 0.06 | 0.50 | 0.01 |  | 0.08 | -0.15 | 0.31 | 0.49 |  |
| Received care:mother absent | -0.30 | -1.19 | 0.60 | 0.52 |  | -0.10 | -1.02 | 0.81 | 0.82 |  | 0.31 | -0.49 | 1.10 | 0.45 |  | -0.14 | -0.95 | 0.67 | 0.74 |  |

**Supplementary Table S8 – Care from maternal aunts/uncles and children’s growth outcomes.** The row ‘care received and mother absent’ shows the effect of receiving high/low care from maternal aunts/uncles on children’s HAZ/WHZ for children who did not live with their mothers. The row ‘care received and mother present’ shows the effect of receiving high/low care from maternal aunts/uncles on children’s HAZ/WHZ for children who lived with their mothers. The rows ‘child’s age’ till ‘received high care from MGP’ are control variables. pH = pHolm.

|  |  | HAZ | | | | | | | |  |  | WHZ | | | | | | | |  |
| --- | --- | --- | --- | --- | --- | --- | --- | --- | --- | --- | --- | --- | --- | --- | --- | --- | --- | --- | --- | --- |
| Caregiver: maternal aunts/uncles | **High-intensity care** | | | |  | **Low-intensity care** | | | |  | **High-intensity care** | | | |  | **Low-intensity care** | | | |  |
| Mother is non-resident | **est.** | **95% CI** | | **p** | **pH** | **est.** | **95% CI** | | **p** | **pH** | **est.** | **95% CI** | | **p** | **pH** | **est.** | **95% CI** | | **p** | **pH** |
| (Intercept) | -1.00 | -1.76 | -0.24 | 0.01 |  | -1.10 | -1.81 | -0.40 | 0.00 |  | 0.79 | 0.11 | 1.46 | 0.02 |  | 0.68 | 0.06 | 1.31 | 0.03 |  |
| Care received | -0.53 | -1.36 | 0.31 | 0.22 | 1.00 | -0.47 | -1.35 | 0.41 | 0.30 | 1.00 | -0.38 | -1.12 | 0.36 | 0.31 | 1.00 | -0.07 | -0.85 | 0.72 | 0.87 | 1.00 |
| Mother present | -0.19 | -0.78 | 0.41 | 0.54 |  | -0.10 | -0.63 | 0.43 | 0.72 |  | 0.01 | -0.52 | 0.54 | 0.98 |  | 0.11 | -0.37 | 0.58 | 0.66 |  |
| Child’s age | -0.07 | -0.16 | 0.02 | 0.11 |  | -0.07 | -0.16 | 0.02 | 0.11 |  | -0.12 | -0.20 | -0.04 | 0.00 |  | -0.12 | -0.20 | -0.04 | 0.00 |  |
| Child's is first child (ref: not first child) | -0.26 | -0.56 | 0.04 | 0.09 |  | -0.25 | -0.55 | 0.05 | 0.10 |  | 0.25 | -0.01 | 0.52 | 0.06 |  | 0.27 | 0.00 | 0.53 | 0.05 |  |
| Food security | -0.01 | -0.03 | 0.00 | 0.07 |  | -0.01 | -0.03 | 0.00 | 0.11 |  | 0.00 | -0.02 | 0.01 | 0.84 |  | 0.00 | -0.02 | 0.01 | 0.85 |  |
| Child was sick (ref: not sick) | 0.04 | -0.22 | 0.31 | 0.75 |  | 0.04 | -0.22 | 0.31 | 0.76 |  | -0.27 | -0.50 | -0.03 | 0.03 |  | -0.25 | -0.49 | -0.01 | 0.04 |  |
| Number under-10s in house | -0.02 | -0.09 | 0.04 | 0.48 |  | -0.02 | -0.08 | 0.04 | 0.50 |  | -0.03 | -0.09 | 0.02 | 0.22 |  | -0.03 | -0.09 | 0.02 | 0.26 |  |
| Urban residence (ref: rural) | 0.12 | -0.12 | 0.36 | 0.31 |  | 0.13 | -0.11 | 0.37 | 0.28 |  | -0.06 | -0.27 | 0.15 | 0.58 |  | -0.06 | -0.28 | 0.15 | 0.56 |  |
| Received high care from MGP (ref: no) | -0.15 | -0.46 | 0.17 | 0.36 |  | -0.20 | -0.52 | 0.12 | 0.22 |  | -0.04 | -0.32 | 0.24 | 0.78 |  | -0.21 | -0.49 | 0.08 | 0.16 |  |
| Received care:mother present | 0.44 | -0.43 | 1.32 | 0.32 |  | 0.34 | -0.60 | 1.28 | 0.48 |  | 0.51 | -0.27 | 1.29 | 0.20 |  | 0.34 | -0.50 | 1.18 | 0.43 |  |
|  |  |  |  |  |  |  |  |  |  |  |  |  |  |  |  |  |  |  |  |  |
| Mother is co-resident | **est.** | **95% CI** | | **p** | **pH** | **est.** | **95% CI** | | **p** | **pH** | **est.** | **95% CI** | | **p** | **pH** | **est.** | **95% CI** | | **p** | **pH** |
| (Intercept) | -1.19 | -1.62 | -0.75 | 0.00 |  | -1.20 | -1.64 | -0.77 | 0.00 |  | 0.79 | 0.40 | 1.18 | 0.00 |  | 0.79 | 0.40 | 1.18 | 0.00 |  |
| Care received | -0.08 | -0.43 | 0.26 | 0.64 | 1.00 | -0.13 | -0.52 | 0.25 | 0.50 | 1.00 | 0.13 | -0.18 | 0.44 | 0.42 | 1.00 | 0.27 | -0.07 | 0.61 | 0.12 | 0.72 |
| Mother absent | 0.19 | -0.41 | 0.78 | 0.54 |  | 0.10 | -0.43 | 0.63 | 0.72 |  | -0.01 | -0.54 | 0.52 | 0.98 |  | -0.11 | -0.58 | 0.37 | 0.66 |  |
| Child’s age | -0.07 | -0.16 | 0.02 | 0.11 |  | -0.07 | -0.16 | 0.02 | 0.11 |  | -0.12 | -0.20 | -0.04 | 0.00 |  | -0.12 | -0.20 | -0.04 | 0.00 |  |
| Child's is first child (ref: not first child) | -0.26 | -0.56 | 0.04 | 0.09 |  | -0.25 | -0.55 | 0.05 | 0.10 |  | 0.25 | -0.01 | 0.52 | 0.06 |  | 0.27 | 0.00 | 0.53 | 0.05 |  |
| Food security | -0.01 | -0.03 | 0.00 | 0.07 |  | -0.01 | -0.03 | 0.00 | 0.11 |  | 0.00 | -0.02 | 0.01 | 0.84 |  | 0.00 | -0.02 | 0.01 | 0.85 |  |
| Child was sick (ref: not sick) | 0.04 | -0.22 | 0.31 | 0.75 |  | 0.04 | -0.22 | 0.31 | 0.76 |  | -0.27 | -0.50 | -0.03 | 0.03 |  | -0.25 | -0.49 | -0.01 | 0.04 |  |
| Number under-10s in house | -0.02 | -0.09 | 0.04 | 0.48 |  | -0.02 | -0.08 | 0.04 | 0.50 |  | -0.03 | -0.09 | 0.02 | 0.22 |  | -0.03 | -0.09 | 0.02 | 0.26 |  |
| Urban residence (ref: rural) | 0.12 | -0.12 | 0.36 | 0.31 |  | 0.13 | -0.11 | 0.37 | 0.28 |  | -0.06 | -0.27 | 0.15 | 0.58 |  | -0.06 | -0.28 | 0.15 | 0.56 |  |
| Received high care from MGP (ref: no) | -0.15 | -0.46 | 0.17 | 0.36 |  | -0.20 | -0.52 | 0.12 | 0.22 |  | -0.04 | -0.32 | 0.24 | 0.78 |  | -0.21 | -0.49 | 0.08 | 0.16 |  |
| Received care:mother absent | -0.44 | -1.32 | 0.43 | 0.32 |  | -0.34 | -1.28 | 0.60 | 0.48 |  | -0.51 | -1.29 | 0.27 | 0.20 |  | -0.34 | -1.18 | 0.50 | 0.43 |  |

**Supplementary Table S9 – Care from paternal aunts/uncles and children’s growth outcomes.** The row ‘care received and mother absent’ shows the effect of receiving high/low care from paternal aunts/uncles on children’s HAZ/WHZ for children who did not live with their mothers. The row ‘care received and mother present’ shows the effect of receiving high/low care from paternal aunts/uncles on children’s HAZ/WHZ for children who lived with their mothers. The rows ‘child’s age’ till ‘received high care from PGP’ are control variables. pH = pHolm.

|  |  | HAZ | | | | | | | |  |  | WHZ | | | | | | | |  |
| --- | --- | --- | --- | --- | --- | --- | --- | --- | --- | --- | --- | --- | --- | --- | --- | --- | --- | --- | --- | --- |
| Caregiver: paternal aunts/uncles | **High-intensity care** | | | |  | **Low-intensity care** | | | |  | **High-intensity care** | | | |  | **Low-intensity care** | | | |  |
| Mother is non-resident | **est.** | **95% CI** | | **p** | **pH** | **est.** | **95% CI** | | **p** | **pH** | **est.** | **95% CI** | | **p** | **pH** | **est.** | **95% CI** | | **p** | **pH** |
| (Intercept) | -1.28 | -1.98 | -0.59 | 0.00 |  | -1.28 | -1.96 | -0.59 | 0.00 |  | 0.59 | -0.02 | 1.21 | 0.06 |  | 0.55 | -0.06 | 1.15 | 0.08 |  |
| Care received | -0.13 | -1.03 | 0.77 | 0.77 | 1.00 | -0.26 | -1.22 | 0.69 | 0.59 | 1.00 | -0.07 | -0.87 | 0.72 | 0.86 | 1.00 | -0.13 | -0.98 | 0.72 | 0.76 | 1.00 |
| Mother present | 0.07 | -0.46 | 0.60 | 0.80 |  | 0.07 | -0.44 | 0.57 | 0.79 |  | 0.19 | -0.28 | 0.66 | 0.43 |  | 0.23 | -0.21 | 0.68 | 0.30 |  |
| Child’s age | -0.07 | -0.16 | 0.02 | 0.12 |  | -0.07 | -0.16 | 0.02 | 0.12 |  | -0.12 | -0.20 | -0.04 | 0.00 |  | -0.12 | -0.20 | -0.04 | 0.00 |  |
| Child's is first child (ref: not first child) | -0.29 | -0.59 | 0.00 | 0.05 |  | -0.30 | -0.59 | 0.00 | 0.05 |  | 0.26 | -0.01 | 0.52 | 0.05 |  | 0.22 | -0.04 | 0.48 | 0.10 |  |
| Food security | -0.01 | -0.03 | 0.00 | 0.07 |  | -0.01 | -0.03 | 0.00 | 0.08 |  | 0.00 | -0.01 | 0.01 | 0.97 |  | 0.00 | -0.02 | 0.01 | 0.83 |  |
| Child was sick (ref: not sick) | 0.03 | -0.24 | 0.30 | 0.83 |  | 0.02 | -0.25 | 0.29 | 0.88 |  | -0.28 | -0.51 | -0.04 | 0.02 |  | -0.27 | -0.51 | -0.03 | 0.02 |  |
| Number under-10s in house | -0.02 | -0.08 | 0.05 | 0.55 |  | -0.02 | -0.09 | 0.04 | 0.50 |  | -0.04 | -0.09 | 0.02 | 0.23 |  | -0.04 | -0.10 | 0.01 | 0.13 |  |
| Urban residence (ref: rural) | 0.10 | -0.14 | 0.35 | 0.39 |  | 0.11 | -0.13 | 0.35 | 0.38 |  | -0.06 | -0.27 | 0.16 | 0.61 |  | -0.04 | -0.26 | 0.17 | 0.70 |  |
| Received high care from PGP (ref: no) | 0.03 | -0.35 | 0.40 | 0.89 |  | 0.07 | -0.31 | 0.45 | 0.70 |  | 0.00 | -0.33 | 0.33 | 0.98 |  | 0.23 | -0.11 | 0.57 | 0.19 |  |
| Received care:mother present | -0.06 | -1.02 | 0.91 | 0.91 |  | -0.11 | -1.18 | 0.96 | 0.83 |  | 0.23 | -0.63 | 1.09 | 0.60 |  | 0.45 | -0.50 | 1.40 | 0.35 |  |
|  |  |  |  |  |  |  |  |  |  |  |  |  |  |  |  |  |  |  |  |  |
| Mother is co-resident | **est.** | **95% CI** | | **p** | **pH** | **est.** | **95% CI** | | **p** | **pH** | **est.** | **95% CI** | | **p** | **pH** | **est.** | **95% CI** | | **p** | **pH** |
| (Intercept) | -1.21 | -1.65 | -0.78 | 0.00 |  | -1.21 | -1.65 | -0.77 | 0.00 |  | 0.78 | 0.40 | 1.17 | 0.00 |  | 0.78 | 0.39 | 1.17 | 0.00 |  |
| Care received | -0.19 | -0.65 | 0.27 | 0.42 | 1.00 | -0.38 | -0.93 | 0.18 | 0.18 | 1.00 | 0.15 | -0.25 | 0.56 | 0.46 | 1.00 | 0.32 | -0.17 | 0.81 | 0.20 | 0.76 |
| Mother absent | -0.07 | -0.60 | 0.46 | 0.80 |  | -0.07 | -0.57 | 0.44 | 0.79 |  | -0.19 | -0.66 | 0.28 | 0.43 |  | -0.23 | -0.68 | 0.21 | 0.30 |  |
| Child’s age | -0.07 | -0.16 | 0.02 | 0.12 |  | -0.07 | -0.16 | 0.02 | 0.12 |  | -0.12 | -0.20 | -0.04 | 0.00 |  | -0.12 | -0.20 | -0.04 | 0.00 |  |
| Child's is first child (ref: not first child) | -0.29 | -0.59 | 0.00 | 0.05 |  | -0.30 | -0.59 | 0.00 | 0.05 |  | 0.26 | -0.01 | 0.52 | 0.05 |  | 0.22 | -0.04 | 0.48 | 0.10 |  |
| Food security | -0.01 | -0.03 | 0.00 | 0.07 |  | -0.01 | -0.03 | 0.00 | 0.08 |  | 0.00 | -0.01 | 0.01 | 0.97 |  | 0.00 | -0.02 | 0.01 | 0.83 |  |
| Child was sick (ref: not sick) | 0.03 | -0.24 | 0.30 | 0.83 |  | 0.02 | -0.25 | 0.29 | 0.88 |  | -0.28 | -0.51 | -0.04 | 0.02 |  | -0.27 | -0.51 | -0.03 | 0.02 |  |
| Number under-10s in house | -0.02 | -0.08 | 0.05 | 0.55 |  | -0.02 | -0.09 | 0.04 | 0.50 |  | -0.04 | -0.09 | 0.02 | 0.23 |  | -0.04 | -0.10 | 0.01 | 0.13 |  |
| Urban residence (ref: rural) | 0.10 | -0.14 | 0.35 | 0.39 |  | 0.11 | -0.13 | 0.35 | 0.38 |  | -0.06 | -0.27 | 0.16 | 0.61 |  | -0.04 | -0.26 | 0.17 | 0.70 |  |
| Received high care from PGP (ref: no) | 0.03 | -0.35 | 0.40 | 0.89 |  | 0.07 | -0.31 | 0.45 | 0.70 |  | 0.00 | -0.33 | 0.33 | 0.98 |  | 0.23 | -0.11 | 0.57 | 0.19 |  |
| Received care:mother absent | 0.06 | -0.91 | 1.02 | 0.91 |  | 0.11 | -0.96 | 1.18 | 0.83 |  | -0.23 | -1.09 | 0.63 | 0.60 |  | -0.45 | -1.40 | 0.50 | 0.35 |  |

**Supplementary Table S10 – Care from distant kin and/or non-kin and children’s growth outcomes.** The row ‘care received and mother absent’ shows the effect of receiving high/low care from distant kin/non-kin on children’s HAZ/WHZ for children who did not live with their mothers. The row ‘care received and mother present’ shows the effect of receiving high/low care from distant kin/non-kin on children’s HAZ/WHZ for children who lived with their mothers. The rows ‘child’s age’ till ‘urban residence’ are control variables. pH = pHolm.

|  |  | HAZ | | | | | | | |  |  | WHZ | | | | | | | |  |
| --- | --- | --- | --- | --- | --- | --- | --- | --- | --- | --- | --- | --- | --- | --- | --- | --- | --- | --- | --- | --- |
| Caregiver: distant kin / non-kin | **High-intensity care** | | | |  | **Low-intensity care** | | | |  | **High-intensity care** | | | |  | **Low-intensity care** | | | |  |
| Mother is non-resident | **est.** | **95% CI** | | **p** | **pH** | **est.** | **95% CI** | | **p** | **pH** | **est.** | **95% CI** | | **p** | **pH** | **est.** | **95% CI** | | **p** | **pH** |
| (Intercept) | -1.38 | -2.21 | -0.55 | 0.00 |  | -1.42 | -2.04 | -0.80 | 0.00 |  | 0.48 | -0.27 | 1.22 | 0.21 |  | 0.67 | 0.12 | 1.23 | 0.02 |  |
| Care received | -0.27 | -1.08 | 0.55 | 0.53 | 1.00 | -0.09 | -0.86 | 0.68 | 0.82 | 1.00 | 0.21 | -0.52 | 0.94 | 0.57 | 1.00 | -0.09 | -0.77 | 0.60 | 0.81 | 1.00 |
| Mother present | 0.16 | -0.59 | 0.91 | 0.67 |  | 0.12 | -0.34 | 0.57 | 0.61 |  | 0.49 | -0.17 | 1.16 | 0.15 |  | 0.23 | -0.18 | 0.63 | 0.27 |  |
| Child’s age | -0.04 | -0.13 | 0.05 | 0.35 |  | -0.06 | -0.15 | 0.02 | 0.14 |  | -0.13 | -0.21 | -0.05 | 0.00 |  | -0.14 | -0.21 | -0.06 | 0.00 |  |
| Food security | -0.01 | -0.03 | 0.00 | 0.13 |  | -0.01 | -0.03 | 0.00 | 0.08 |  | 0.00 | -0.02 | 0.01 | 0.86 |  | 0.00 | -0.02 | 0.01 | 0.88 |  |
| Child was sick (ref: not sick) | 0.02 | -0.24 | 0.28 | 0.88 |  | 0.03 | -0.23 | 0.29 | 0.81 |  | -0.32 | -0.55 | -0.09 | 0.01 |  | -0.31 | -0.54 | -0.08 | 0.01 |  |
| Number under-10s in house | -0.01 | -0.07 | 0.05 | 0.67 |  | -0.02 | -0.08 | 0.04 | 0.55 |  | -0.04 | -0.09 | 0.02 | 0.20 |  | -0.03 | -0.09 | 0.02 | 0.23 |  |
| Urban residence (ref: rural) | 0.10 | -0.13 | 0.34 | 0.40 |  | 0.05 | -0.18 | 0.29 | 0.65 |  | -0.02 | -0.23 | 0.19 | 0.85 |  | 0.00 | -0.22 | 0.21 | 0.97 |  |
| Received care:mother present | 0.00 | -0.86 | 0.86 | 1.00 |  | 0.19 | -0.65 | 1.02 | 0.66 |  | -0.37 | -1.13 | 0.40 | 0.34 |  | -0.11 | -0.85 | 0.64 | 0.78 |  |
|  |  |  |  |  |  |  |  |  |  |  |  |  |  |  |  |  |  |  |  |  |
| Mother is co-resident | **est.** | **95% CI** | | **p** | **pH** | **est.** | **95% CI** | | **p** | **pH** | **est.** | **95% CI** | | **p** | **pH** | **est.** | **95% CI** | | **p** | **pH** |
| (Intercept) | -1.22 | -1.65 | -0.79 | 0.00 |  | -1.30 | -1.72 | -0.88 | 0.00 |  | 0.97 | 0.59 | 1.35 | 0.00 |  | 0.90 | 0.52 | 1.28 | 0.00 |  |
| Care received | **-0.27** | **-0.53** | **0.00** | **0.05** | 0.35 | 0.10 | -0.22 | 0.42 | 0.55 | 1.00 | -0.16 | -0.39 | 0.08 | 0.18 | 1.00 | -0.19 | -0.48 | 0.09 | 0.19 | 0.76 |
| Mother absent | -0.16 | -0.91 | 0.59 | 0.67 |  | -0.12 | -0.57 | 0.34 | 0.61 |  | -0.49 | -1.16 | 0.17 | 0.15 |  | -0.23 | -0.63 | 0.18 | 0.27 |  |
| Child’s age | -0.04 | -0.13 | 0.05 | 0.35 |  | -0.06 | -0.15 | 0.02 | 0.14 |  | -0.13 | -0.21 | -0.05 | 0.00 |  | -0.14 | -0.21 | -0.06 | 0.00 |  |
| Food security | -0.01 | -0.03 | 0.00 | 0.13 |  | -0.01 | -0.03 | 0.00 | 0.08 |  | 0.00 | -0.02 | 0.01 | 0.86 |  | 0.00 | -0.02 | 0.01 | 0.88 |  |
| Child was sick (ref: not sick) | 0.02 | -0.24 | 0.28 | 0.88 |  | 0.03 | -0.23 | 0.29 | 0.81 |  | -0.32 | -0.55 | -0.09 | 0.01 |  | -0.31 | -0.54 | -0.08 | 0.01 |  |
| Number under-10s in house | -0.01 | -0.07 | 0.05 | 0.67 |  | -0.02 | -0.08 | 0.04 | 0.55 |  | -0.04 | -0.09 | 0.02 | 0.20 |  | -0.03 | -0.09 | 0.02 | 0.23 |  |
| Urban residence (ref: rural) | 0.10 | -0.13 | 0.34 | 0.40 |  | 0.05 | -0.18 | 0.29 | 0.65 |  | -0.02 | -0.23 | 0.19 | 0.85 |  | 0.00 | -0.22 | 0.21 | 0.97 |  |
| Received care:mother absent | 0.00 | -0.86 | 0.86 | 1.00 |  | -0.19 | -1.02 | 0.65 | 0.66 |  | 0.37 | -0.40 | 1.13 | 0.34 |  | 0.11 | -0.64 | 0.85 | 0.78 |  |

*Differences in receipt of allomaternal care between sick and healthy children*

**
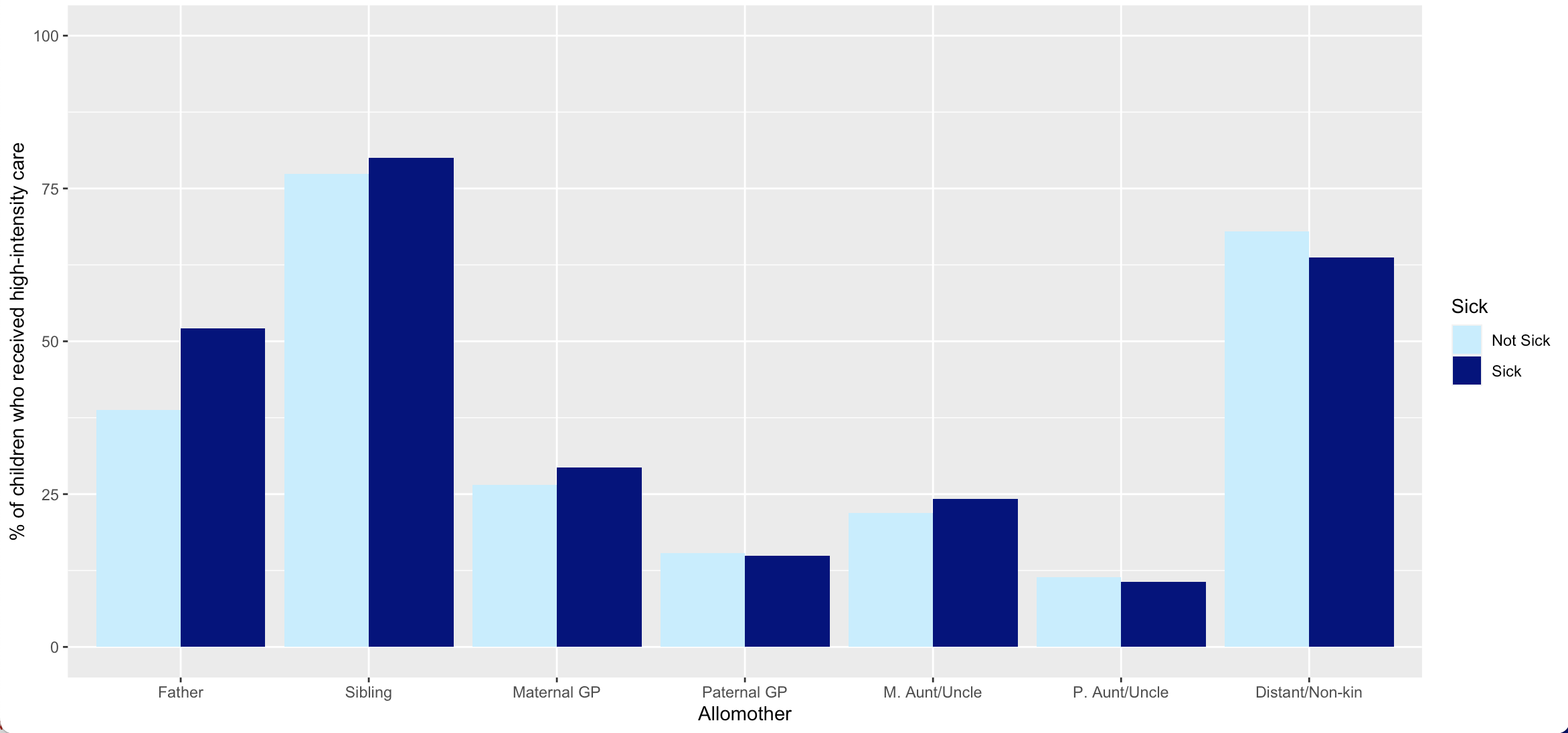
**

**Figure S4 –** Proportion of children who received high-intensity care from each allomother by their sick status (sick on the left-hand bars in light blue, not sick on the right-hand bars in dark blue). This reveals that allomothering did not vary by sickness status, except from for fathers. To test this further, we ran the father care models restricting the data to children who had not been sick (n=593); these children would all have received a 0 for the care type ‘sick’. When restricted to not sick children, the models show the same results as when the full dataset are used. That is, receiving high intensity care from fathers is associated with a higher HAZ score for children with non-resident mothers (effect size = 1.72, p-value = 0.05, 95% CI = 0.39-3.06); and a higher WHZ score for children with co-resident mothers (effect size = 0.36, p-value = 0.01, 95% CI = 0.11-0.61).
